# Potential Impacts of Energy and Vehicle Transformation Through 2050 on Oxidative Stress‐Inducing PM_2.5_ Metals Concentration in Japan

**DOI:** 10.1029/2023GH000789

**Published:** 2023-10-13

**Authors:** Satoko Kayaba, Mizuo Kajino

**Affiliations:** ^1^ Graduate School of Science and Technology University of Tsukuba Tsukuba Japan; ^2^ Meteorological Research Institute Japan Meteorological Agency Tsukuba Japan; ^3^ Faculty of Life and Environmental Sciences University of Tsukuba Tsukuba Japan

**Keywords:** transition metals, oxidative stress, non‐exhaust PM, renewable energy, electric vehicle, air quality

## Abstract

The impacts of renewable energy shifting, passenger car electrification, and lightweighting through 2050 on the atmospheric concentrations of PM_2.5_ total mass and oxidative stress‐inducing metals (PM_2.5_‐Fe, Cu, and Zn) in Japan were evaluated using a regional meteorology–chemistry model. The surface concentrations of PM_2.5_ total mass, Fe, Cu, and Zn in the urban area decreased by 8%, 13%, 18%, and 5%, respectively. Battery electric vehicles (BEVs) have been considered to have no advantage in terms of non‐exhaust PM emissions by previous studies. This is because the disadvantages (heavier weight increases tire wear, road wear, and resuspention) offset the advantages (regenerative braking system (RBS) reduces brake wear). However, the future lightweighting of drive battery and body frame were estimated to reduce all non‐exhaust PM. Passenger car electrification only reduced PM_2.5_ concentration by 2%. However, Fe and Cu concentrations were more reduced (−8% and −13%, respectively) because they have high brake wear‐derived and significantly reflects the benefits of BEV's RBS. The water‐soluble fraction concentration of metals (induces oxidative stress in the body) was estimated based on aerosol acidity. The reduction of SO_x_, NO_x_, and NH_3_ emissions from on‐road and thermal power plants slightly changed the aerosol acidity (pH ± 0.2). However, it had a negligible effect on water‐soluble metal concentrations (maximum +2% for Fe and +0.5% for Cu and Zn). Therefore, the metal emissions reduction was more important than gaseous pollutants in decreasing the water‐soluble metals that induces respiratory oxidative stress and passenger car electrification and lightweighting were effective means of achieving this.

## Key Points

- Vehicle electrification effectively reduced the concentrations of PM_2.5_‐Fe and Cu, which are toxic to respiratory oxidative stress
- The lightweighting of battery electric vehicles reduced all non‐exhaust PM from tire, brake, road wear, and resuspension
- Changes in aerosol acidity due to gaseous pollutants reduction had little effect on the water‐solubility of metals

## 1 Introduction

There is a strong association between the dry mass of particulate matter with aerodynamic diameters of 2.5 μm or less (PM_2.5_) and human health effects (Pope & Dockery, 2006; Valavanidis et al., 2008). However, PM_2.5_ consists of various different chemical components, and their toxicities vary greatly depending on the components. Highly toxic components may contribute significantly to the total toxicity of PM_2.5_, even if their mass concentrations are low. It is desirable to take this into consideration when conducting emission source control.

Metal components in PM induce oxidative stress by consuming antioxidants and producing reactive oxygen species (ROS) such as O_2_^−^, H_2_O_2_, HO_2_, and OH in the body (Bates et al., 2019; Lakey et al., 2016; Shiraiwa et al., 2017). Oxidative stress leads to respiratory inflammation, asthma, chronic obstructive pulmonary disease (COPD), and other diseases (U.S. EPA, 2019). Transition metals such as Fe and Cu have redox active and catalyze the production of ROS through the redox cycle. It has been reported that Cu has the highest OPDTT (consumption rate of DTT, a reducing agent, which mimics the consumption rate of antioxidants in the body) of all transition metals (Charrier & Anastasio, 2012). Fe efficiently produces OH, the most oxidizing and toxic of all the ROS, through Fenton reactions in the body (Charrier & Anastasio, 2011; Valavanidis et al., 2000, 2008). Zn does not have redox active, but causes oxidative stress through biological mechanisms (Gottipolu et al., 2008; Samet et al., 2020; Wu et al., 2013). For example, Zn^2+^ binds to the proton channel of cytochrome c oxidase (CCO) and inactivates CCO, which is expected to result in increased production of O_2_^−^ from complexes I and III (Handy & Loscalzo, 2012). Since all of the above ROS production processes are caused by free ionized metals in the lung fluid, the solubility of metals is an important factor in toxicity (Adamson et al., 2000; Costa & Dreher, 1997). The increase of aerosol acidity due to sulfuric acid or nitric acid affects the solubility of metals (Fang et al., 2017; Meskhidze et al., 2003; Oakes et al., 2012; Shahpoury et al., 2021; Yang & Weber, 2022).

Based on the Paris Agreement, many countries have declared their future carbon neutrality. Japan has also set a goal of becoming carbon neutral by 2050, and in order to achieve it, renewable energy and next‐generation vehicles such as battery electric vehicles (BEVs) are being promoted (Cabinet Office of Japan, 2021; METI, 2021; MLIT, 2018). It would change not only greenhouse gases, but also air pollutant emissions. In road transport sector, vehicle exhaust emissions have been significantly improved so far. On the other hand, the contribution of “non‐exhaust PM” emissions such as brake, tire, and road surface wear is becoming more significant (CEC, 2022; OECD, 2020; Vanherle et al., 2021). Non‐exhaust PM is one of the major emission sources of metals. Fe, Cu, Ba, and Sb are the main metal components of brake wear PM (Grigoratos & Martini, 2014). The most abundant metal in tire treads is Zn, which is added as a vulcanizing agent and accounts for approximately 1% of the PM_2.5_ size mass of tire wear particles (Blok, 2005; Grigoratos & Martini, 2014; Smolders & Degryse, 2002). Several reports and review articles have pointed out the risk of non‐exhaust PM‐derived metals causing the above health effects (Fussell et al., 2022; Grigoratos & Martini, 2014). However, regulations for non‐exhaust PM are currently limited to only a few regions (OECD, 2020) and are currently in the process of standardizing emission estimates (EMEP/EEA, 2019) and considering measures.

BEVs have advantages and disadvantages in terms of non‐exhaust PM emission. Compared to internal combustion engine vehicles (ICEV), the increased vehicle weight due to the drive battery increases non‐exhaust PM (Timmers & Achten, 2016), while regenerative braking system (RBS) reduces the use of conventional friction brakes and decrease brake wear. Several studies have estimated that these advantages and disadvantages offset each other and that BEVs do not provide much benefit in terms of total PM emissions, and this seems to be the consensus (Alam et al., 2018; Beddows & Harrison, 2021; Fussell et al., 2022; Mehlig et al., 2021; OECD, 2020; Sisani et al., 2022; Timmers & Achten, 2016). For example, OECD (2020) estimated that, for lightweight BEVs (range of 100 miles), the benefits of the RBS would dominate the effect of vehicle weight increase, reducing PM_2.5_ by approximately 11%–13%, while heavy BEVs (range of 300 miles) would increase PM_2.5_ by 3%–8% because the significant weight increase would mainly increase tire wear. Beddows and Harrison (2021) reported that BEVs may not reduce total PM emissions more than ICEVs in highway driving with less braking.

However, the relative weight ratios of BEVs to ICEVs in all these previous studies were based on current assumptions. In reality, it is expected that vehicle lightweighting technologies, including batteries for BEVs, will advance in the future (Kelly et al., 2015; Moawad et al., 2011, 2016), but no studies have evaluated considering this. In addition, several studies have evaluated the impact of the penetration of next‐generation vehicles on atmospheric PM2.5 total mass concentration using chemical transport modeling (CTM) (Ke et al., 2017; Li et al., 2016; Nopmongcol et al., 2017; Pan et al., 2019; Schnell et al., 2019; Soret et al., 2014; Tessum et al., 2014), but no studies have focused on especially high toxic components to human health. In this study, the impacts of changes in primary emissions associated with the renewable energy shifting, passenger car electrification, and lightweighting through 2050 on not only the atmospheric concentration of PM_2.5_ total mass, but also those of water‐soluble fraction of metal species (Fe, Cu, and Zn) were evaluated using CTM. These metal species were chosen because they induce oxidative stress in the respiratory system and are major in vehicle non‐exhaust PM. Because the CTM used in this study can not directly estimate the concentration of water‐soluble fraction of metals, it was estimated from aerosol acidity. In Section 2, the methodology is explained, including an overview of the CTM, assumptions for sensitivity experiments, and observation data for model evaluation. In Section 3, the reproducibility of the model is verified first. Then, the impacts of renewable energy shifting, passenger car electrification, and lightweighting on the concentrations of the PM_2.5_ total mass, Fe, Cu, and Zn and aerosol pH are evaluated. By integrating these results, changes in water‐soluble metal concentrations are discussed. In Section 4, we present conclusions and discuss future work.

## 2 Materials and Methods

### 2.1 Regional Meteorology–Chemistry Model

**Figure 1:**
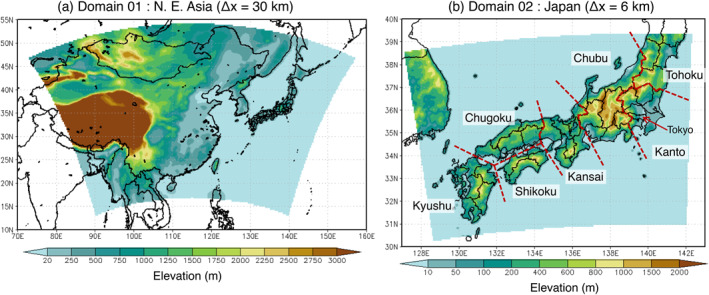
Model domains in this study. (a) Terrestrial elevations of domain 01 (Northeast Asia, Δ*x* = 30 km) and (b) same as (a) but for domain 02 (Japan, Δ*x* = 6 km).

A regional‐scale offline‐coupled nonhydrostatic meteorology–chemistry model (NHM‐Chem) (full chemistry version; Kajino et al., 2019a, 2021 and transition metal version; Kajino et al., 2020) was used in this study. Detail descriptions are summarized in Table S1 of the Supporting Information S1. Figure 1 shows the model calculation domains. The mother domain (domain 01) covered the Northeast Asian region and was calculated with Δ*x* = 30 km. The nested domain (domain 02) covered Japan from Kyushu to Tohoku regions with Δ*x* = 6 km. NHM‐Chem does not implement two‐way nested domain system so that there is no feedback from nested to mother domains considered. The vertical layer involved 40 layers up to an altitude of approximately 20 km in both domains. Hereafter, the model simulation results were of the lowest level (from ground surface to approximately 50 m) unless otherwise noted. The calculation period was from 1 January 2015, to 31 December 2015; the simulation began on 26 December 2014, with a spin‐up period of 5 days.

**Figure 2:**
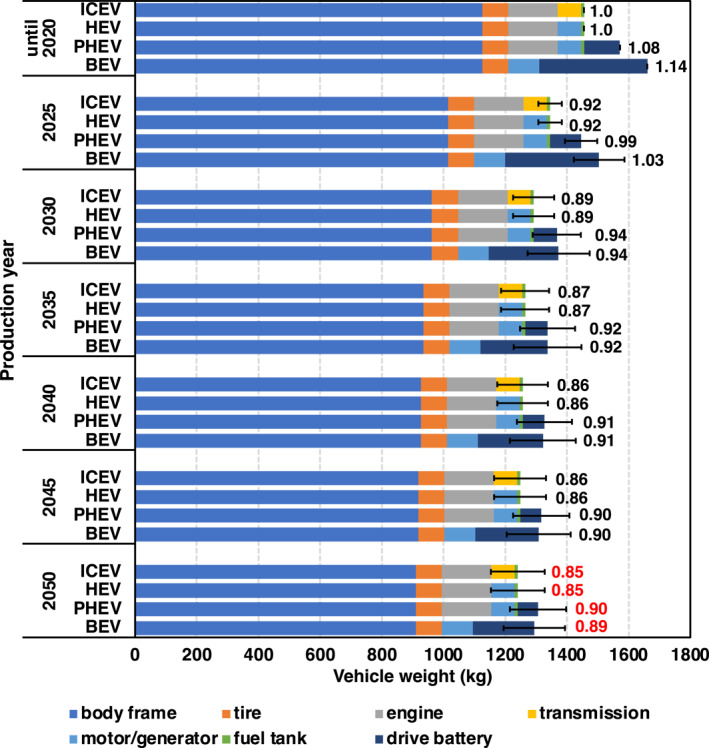
Vehicle weight transition through 2050. The numbers shown along with the bars represent the values relative to ICEVs produced until 2020. The error bars indicate the uncertainty in the degree of the technological progress of lightweighting (low and high). The weight of each component in the base year (until 2020) was based on literature values derived using Autonomie (Moawad et al., 2016). Only the glider and the drive battery were assumed to be lightweight (weight reduction rates are shown in Tables S4 and S5 of the Supporting Information S1, respectively), while the weights of other components remained unchanged. Note that the on‐board battery capacity (i.e., cruising range) is assumed to be fixed.

First, the emission inventories used for gas and particle calculations for the full chemistry version model are described. For Northeast Asian anthropogenic emissions, REAS v3.2.1 (minor change in December 2021 from v3.2 (Kurokawa & Ohara, 2020a, 2020b), 0.25° × 0.25°, base year = 2015) and for Japan, PM2.5EI (Morikawa, 2017, 1 km × 1 km, base year = 2012) were used. NO_x_ emissions were allocated 9:1 to NO and NO_2_ for both REAS v3.2.1 and PM2.5EI. Taking chimney elevation into account, emissions from industries and power plants were distributed in this study from 0 to 300 m above the ground level. We assumed the following for the original database of PM2.5EI in this study.

- In PM2.5EI, since there is no information on the mass fraction of “brake wear” in “road dust (including brake wear),” it was assumed to be 0.35 based on the value calculated by EMEP/EEA (2019). The EMEP/EEA air pollutant emissions inventory guidebook (2019) was published by the European Environment Agency (EEA) and supports the reporting of emissions data under the UNECE convention on long‐range transboundary air pollution (CLRTAP) and the EU national emissions control directives. In this study, the remainder of “road dust (including brake wear)” minus “brake wear” is called “road wear and resuspension.”
- In PM2.5EI, non‐exhaust PM emissions from automobiles are not classified by particle size (those from other sources are classified as PM_2.5_ or PM_10_). In this study, the PM_2.5_/PM_10_ mass fraction of non‐exhaust PM was assumed to be 0.35 based on hearing information from JCAP/JATOP and the estimated data of EMEP/EEA (2019). EMEP/EEA (2019) estimated the PM_2.5_ fraction for tire wear, brake wear and road surface wear to be 0.42, 0.39, and 0.27, respectively, their mean is approximately 0.35. NHM‐Chem assumes a log‐normal distribution for the PM particle size distribution at emission. By setting the parameter of number‐equivalent geometric mean dry diameter = 0.80 μm (number‐equivalent aerodynamic mean dry diameter = 1.13 μm), and standard deviation = 1.8, the aerodynamic PM_2.5_ fraction is approximately 0.35.
- The PM2.5EI data we had available did not provide the information on the mass fractions of BC and OC in PM_2.5_ and PM_10_ emissions, we applied the corresponding REAS v3.2.1 mass fractions for each sector of PM2.5EI. The remaining primary PM emissions, excluding BC and OC, were defined as inert unidentified components (UIDs).
- For domain 2 over Japan, the ship emissions from EAGrid (Fukui et al., 2014; Kannari et al., 2007) were added because PM2.5EI does not provide ship emissions.

GFED v4 (Giglio et al., 2013a, 2013b) was used for biomass burning emissions and JMA data was used for volcanic SO_2_ emissions for both domains. Biogenic nonmethane volatile organic compound (NMVOCs) emissions were calculated inline based on MEGAN v2 (Guenther et al., 2006) as a function of temperature and solar radiation, simulated by the meteorological model.

The transition metal version model simulated 10 metals (Cu, Mn, Co, V, Ni, Pb, Fe, Zn, Cd, and Cr) in three categories (anthropogenic PM_2.5_ metals, anthropogenic PM_10_ metals, and Asian mineral dust metals). The anthropogenic emission inventory used for the simulations of PM_2.5_ and PM_10_ metals was TMI‐Asia/Japan v1.1, developed in this study. The previous version (v1.0; Kajino et al., 2020) contained considerable discrepancies between simulated and observed metal concentrations, especially for Cu and Zn, which were substantially improved in this revision. TMI‐Japan v1.0 considered metal emissions from brake and tire wear but did not considered those from road wear and resuspension. They were newly added in v1.1. The details of the revisions from v1.0 to v1.1 are described in Text S1, Figures S1–S4, and Tables S1–S3 of the Supporting Information S1. TMI‐Asia/Japan were developed by multiplying sector‐specific PM_2.5_ or PM_10_ emission estimates from REAS v2 (Kurokawa et al., 2013, 0.25° × 0.25°, base year = 2008) and PM2.5EI by metal content, respectively. However, in TMI‐Japan, brake, tire, and railway‐derived metals were based on PM emission estimates from EAGrid (1 km × 1 km, base year = 2010). The metal content of PM by sector is an average of several literature values registered in SPECIATE v4.4 provided by the U.S. EPA. The list of metal content by sector used in TMI‐Asia/Japan v1.1 is available from Kayaba (2023b). The metal emissions in Asian mineral dust particles were diagnosed from the simulated dust mass concentration and the metal profiles of the Certified Reference Material of the National Institute for Environmental Studies (NIES CRM No. 30; Gobi Kosa) (Nishikawa et al., 2013).

For the initial and boundary conditions for the NHM (meteorological model part of NHM‐Chem), we used a 6‐hourly JRA‐55 global reanalysis data set (Kobayashi et al., 2015) for domain 01 and 3‐hourly JMA's Meso‐Regional Objective Analysis (MA) for domain 02 (available at https://www.jma.go.jp/jma/jma-eng/jma-center/nwp/nwp-top.htm, last accessed: 20 January 2023). For the large‐wave components of horizontal momentum and potential temperature (wavelengths >1,000 km), spectral nudges above a 7‐km altitude were applied, and the weighting factor was set to 0.06. For the CTM part of NHM‐Chem, monthly climatological values (10‐years averages for the global models MRI‐CCM2 and MASINGAR‐mk2 from 2003 to 2013) were used for the initial and boundary concentrations in domain 01, and the results of domain 01 were used for domain 02. The input/output time interval of CTM was 1 hr.

### 2.2 Aerosol pH Calculation

The aerosol pH discussed in Section 3.2.3 was derived using ISORROPIA‐II (Fountoukis & Nenes, 2007). ISORROPIA‐II can simulate the thermodynamic equilibrium of water‐soluble inorganic ions and calculate the pH of aerosol particles in the equilibrium state. ISORROPIA‐II was implemented in NHM‐Chem for the calculation of the condensation of HNO_3_, NH_3_, HCl, and H_2_O (Kajino et al., 2021), but aerosol pH was not dynamically solved. In addition, ISORROPIA‐II in NHM‐Chem solves the aerosol thermodynamics of each aerosol category but does not solve the aerosol pH of bulk submicron aerosols discussed in the study. Therefore, a standalone ISORROPIA‐II model was used to diagnose the aerosol pH after the NHM‐Chem simulation. In addition, sensitivity tests of Equations (2), (3), (4) were only feasible using this standalone model.

The PM_2.5_ particle size aerosol pH was derived as follows. First, the forward mode of ISORROPIA‐II was run using hourly NHM‐Chem calculation results as input data; the mass concentrations (mol m^−3^) of K^+^, Ca^2+^, Mg^2+^, NH^4+^, Na^+^, SO_4_^2−^, NO_3_^−^, and Cl^−^ in the submicron category (aitken, soot‐free accumulation, and soot containing accumulation modes), relative humidity (RH), and temperature. The output of the 1‐hr aerosol liquid water content (LWC) and hydronium ion concentration data for each model grid were monthly averaged and applied to the following Equation 1 for defining monthly averaged aerosol pH:

(1) $$\text{pH}={-\log}_{10}\left(\gamma_{H^{+}}\cdot H_{\text{aq}}^{+}\right)={-\log}_{10}\left(\frac{{1000\gamma }_{H^{+}}\cdot H_{\text{air}}^{+}}{\text{LWC}}\right)$$

where $\gamma_{H^{+}}$ is the activity coefficient of hydronium ions (assumed = 1), $H_{\text{aq}}^{+}$ is the concentration of hydronium ions in the aerosol water phase (mol L^−1^), $H_{\text{air}}^{+}$ (μg m^−3^) is the concentration of hydronium ions per air volume, and LWC (μg m^−3^) is the water concentration of aerosol particles. However, only 1‐hr data corresponding to 20% < RH < 95% were used for the monthly average. Data with RH < 20% were excluded because the aerosol was unlikely to be in a liquid state and the activity coefficient of hydronium ions in the aerosol water phase was highly uncertain at the case of high concentrations under low RH conditions (Fountoukis et al., 2009; Guo et al., 2016). The LWC increases exponentially with increasing RH due to the hygroscopicity of NH_4_NO_3_ and (NH_4_)_2_SO_4_ (Kitamori et al., 2009). Data with RH > 95% were excluded because the uncertainty in RH could significantly increase the uncertainty in LWC and aerosol pH (Guo et al., 2015, 2016). The LWC mainly depends on hygroscopic inorganic species, such as sulfate. Organics have relatively low hygroscopicity, so their effect on aerosol pH is small and can be negligible (Guo et al., 2015; Pye et al., 2018; Vasilakos et al., 2018). Similar to many other studies (Ding et al., 2019; Lawal et al., 2018; Paglione et al., 2021), this study did not consider the impact of organic matter on aerosol pH. Instead, the uncertainty in aerosol pH due to not considering organic matter is described in Text S2 and Figure S5 of the Supporting Information S1.

As described above, aerosol pH depends on both $H_{\text{air}}^{+}$ and LWC concentration. Acidic substances such as H_2_SO_4_ and HNO_3_ increase $H_{\text{air}}^{+}$, while basic substances such as NH_3_ decrease $H_{\text{air}}^{+}$ in aerosols (hereafter referred to as the $H_{\text{air}}^{+}$ process). The increase in water‐soluble aerosols increases the LWC and decreases the pH (hereafter referred to as the LWC process). The net pH sensitivity (ΔpH_NET_) is equal to the sum of the pH sensitivity of the $H_{\text{air}}^{+}$ process ($\Delta {\text{pH}}_{H^{+}}$) and that of the LWC process (ΔpH_LWC_).

The respective sensitivities can be derived by Equations (2), (3), (4):

(2) $$\Delta {\text{pH}}_{\text{NET}}={-\log}_{10}\left(\frac{{1000\gamma }_{H^{+}}\cdot {H_{\text{air}}^{+}}_{\text{sens}}}{{\text{LWC}}_{\text{sens}}}\right)+\log_{10}\left(\frac{{1000\gamma }_{H^{+}}\cdot {H_{\text{air}}^{+}}_{\text{cntrl}}}{{\text{LWC}}_{\text{cntrl}}}\right)$$

(3) $$\Delta {\text{pH}}_{H^{+}}={-\log}_{10}\left(\frac{{1000\gamma }_{H^{+}}\cdot {H_{\text{air}}^{+}}_{\text{sens}}}{{\text{LWC}}_{\text{cntrl}}}\right)+\log_{10}\left(\frac{{1000\gamma }_{H^{+}}\cdot {H_{\text{air}}^{+}}_{\text{cntrl}}}{{\text{LWC}}_{\text{cntrl}}}\right)$$

(4) $$\Delta {\text{pH}}_{\text{LWC}}={-\log}_{10}\left(\frac{{1000\gamma }_{H^{+}}\cdot {H_{\text{air}}^{+}}_{\text{cntrl}}}{{\text{LWC}}_{\text{sens}}}\right)+\log_{10}\left(\frac{{1000\gamma }_{H^{+}}\cdot {H_{\text{air}}^{+}}_{\text{cntrl}}}{{\text{LWC}}_{\text{cntrl}}}\right)$$

where ${H_{\text{air}}^{+}}_{\text{cntrl}}$ and LWC_cntrl_ are the reference $H_{\text{air}}^{+}$ and LWC concentrations (μg m^−3^), and ${H_{\text{air}}^{+}}_{\text{sens}}$ and LWC_sens_ are those of sensitivity.

### 2.3 Model Experiment Cases and Parameter Setting

#### 2.3.1 Model Experiment Cases

A base experiment and the following three sensitivity experiments were conducted in this study. The emissions for each sensitivity experiment were determined by scaling the base experiment by the coefficients shown in Table 1. Also, Table 2 shows the assumptions that were changed and unchanged from the BESE experiment. Our previous study (Kayaba & Kajino, 2023), which estimated the impact of the BEV shift of all passenger vehicles on surface O_3_ concentration, did not consider the future scenarios of changes in vehicle type mix or power supply mix. However, in this study, we developed detailed future scenarios based on the Japanese government targets. The coefficients were derived based on the estimated trends in vehicle exhaust and non‐exhaust emissions, and gasoline and electricity demands through 2050. Details of the derivation methods are described in Sections 2.3.2–2.3.4.

1. BASE experiment.

**Table 1:** Ratios of Emission Factors for the Sensitivity Experiments (2050R, 2050R&E, and 2050R&E&L) to BASE Experiment

Emission source	Species		BASE	2050R	2050R&E	2050R&E&L
	Particle pollutants	Gaseous pollutants				
Passenger car exhaust	PM_2.5_, Fe, Cu, Zn	SO_x_, NO_x_, NH_3_, NMVOCs^a^, CO	1	1	0.31^b^	0.31^b^
Passenger car evaporative	–	NMVOCs^a^	1	1	0.31^b^	0.31^b^
Passenger car tire wear	PM_2.5_, Zn	–	1	1	1.09^c^	0.89^c^
Passenger car road wear & resuspension	PM_2.5_, Fe, Cu, Zn	–	1	1	1.09^c^	0.89^c^
Passenger car Brake wear	PM_2.5_, Fe, Cu, Zn	–	1	1	0.67^d^	0.55^d^
Thermal power plant	PM_2.5_, Fe, Cu, Zn	SO_x_, NO_x_, NH_3_, NMVOCs^a^, CO	1	0.18^e^	0.19^e^	0.18^e^
Gas station	–	NMVOCs^a^	1	1	0.41^f^	0.33^f^

^a^ Secondary organic aerosol (SOA) formation was not included in the simulations. The changes in NMVOC emissions affect changes in oxidant concentrations such as O_3_, OH, and H_2_O_2_, and the associated changes in secondary inorganic aerosol formation.

^b^ Passenger car electrification was considered (Figure 3d).

^c^ Passenger car electrification and lightweighting were considered (Figure 3b).

^d^ Passenger car electrification, lightweighting, and effect of BEV's RBS were considered (Figure 3c).

^e^ The reduction of thermal power plants was considered (Figure S8b in Supporting Information S1). No increase in thermal power plant emissions was assumed because it was assumed that the additional electricity demand for charging BEVs and PHEVs (Figure 3e) could be covered by the surplus electricity obtained solar power generation.

^f^ The reduction of gasoline consumption by passenger car (80% of total consumption) was assumed (Figure 3f).

**Table 2:** Assumptions That Were Changed or Unchanged in the 2050R&E&L Experiment Compared to the BASE Experiment

Sector	Changed	Unchanged
Vehicle transport	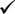 Vehicle type mix (passenger car)	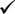 Assumption of truck, bus and motorcycle
	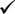 Vehicle lightweighting (passenger car)	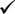 Total traffic volume
	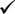 Energy consumption (passenger car)	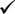 Total vehicle stock
Power plant	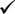 Power supply mix	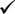 Electricity demand excluding BEV and PHEV charging
	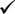 Additional electricity demand for BEV and PHEV charging	
Stationary NMVOCs	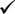 Gasoline fuel demand at gas stations	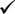 Assumption of other stationary NMVOCs source
Other sectors^a^	–	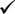 All assumptions

^a^ Industry, domestic, aviation, navigation, railway, off‐road vehicle, and field‐burning.

The simulation period was the whole year 2015, and the base year emissions of inventories were used for the simulation (Table S1 in Supporting Information S1).

2 2050R experiment.

This scenario assumed the penetration of renewable energy, taking into account changes in the power supply mix through 2050. The emissions from power plants were reduced considering the decrease in thermal power generation.

3 2050R&E experiment.

This scenario assumed passenger car electrification (without lightweighting) through 2050 in addition to (2). Changes in exhaust and non‐exhaust emissions due to changes in vehicle type mix (ICEV, hybrid electric vehicle (HEV), PHEV, and BEV) were considered. To assess the impact of passenger car electrification, the assumptions for heavy‐duty vehicles were not changed. Also, the total number of vehicles owned and the volume of traffic were not changed to evaluate the sensitivity of changes in emission factors. The additional electricity demand for charging BEVs and PHEVs was estimated to be mostly covered by solar surpluses, although not completely, resulting in a slight increase in power plant emissions of 1% from (2) (Text S5 in Supporting Information S1). NMVOCs emissions from gas stations were reduced due to the reduced demand for gasoline.

4 2050R&E&L experiment.

This scenario considered passenger car lightweighting through 2050 in addition to (3). Non‐exhaust emissions were reduced from (3) due to vehicle weight reduction. It was assumed that exhaust performance would not change due to lightweighting. No increase in power plant emissions was assumed (same as (2)), because the additional electricity demand for charging BEVs and PHEVs was estimated to be lower than that in (3) because of the lower electricity consumption due to lightweighting, which can be covered by the surplus of solar power. NMVOCs emissions from gas stations were further reduced compared to that in (3) due to the improved of energy consumption by vehicle lightweighting.

#### 2.3.2 Passenger Car Lightweighting Through 2050

Figure 2 shows the change in vehicle weight for each vehicle type through 2050. For each of the four vehicle types (ICEV, HEV, PHEV, and BEV), the weights of six components (body frame, tire, engine, transmission, motor/generator, fuel tank, and drive battery) are combined. Each component weight was referenced to calculations conducted by Autonomie, a vehicle simulation tool developed by the Argonne National Laboratory (ANL) of the U.S. Department of Energy (Islam et al., 2020; Moawad et al., 2016). Autonomie can evaluate vehicle weight, fuel consumption, performance, and cost for various vehicle classes (mini, medium, small sport utility vehicles (SUV), medium SUV, and pickup truck) and vehicle types (ICEV, HEV, PHEV, BEV, and fuel‐cell electric vehicle (FCV)). In this study, only the body frame and the drive battery were assumed to be lightweight through 2050 (their reduction rates are shown in Tables S4 and S5 of the Supporting Information S1, respectively). The lightweighting of body frames will be achieved relatively early in the future by alternative materials, such as high‐strength low‐alloy steels and aluminum. Compared to 2020, approximately −10% will be achieved by 2025, followed by a gradual decrease, with a lightweighting of approximately 20% in 2050 (Table S4 in Supporting Information S1). As the energy density of the battery increases, the weight per unit capacity decreases for the fixed driving range. In this study, we assumed that even if the battery per unit capacity becomes lighter, the on‐board battery capacity (i.e., cruising range) will remain constant through 2050. Then, the battery weight is expected to be approximately 43% lighter in 2050 than in 2020 (Table S5 in Supporting Information S1). The battery weight of the PHEV was assumed to be one third of that of the BEV in this study. The weights of other components such as the engine and motor/generator were assumed to be unchanged. Until 2020, BEVs were net 14% heavier than ICEVs due to their batteries. By 2050, ICEVs and HEVs will be 15% lighter compared to those in 2020 due to the lightweighting of the body frame. PHEVs and BEVs have a larger lightweight ratio than ICEVs and HEVs because of the reduction in battery weight in addition to the reduction in body frame. After 2030, BEVs and PHEVs will be lighter than ICEVs produced until 2020. By 2050, the weight difference between vehicle types will be smaller, with a +5% relative weight difference for BEVs compared to ICEVs.

#### 2.3.3 Vehicle Type Mix Through 2050

**Figure 3:**
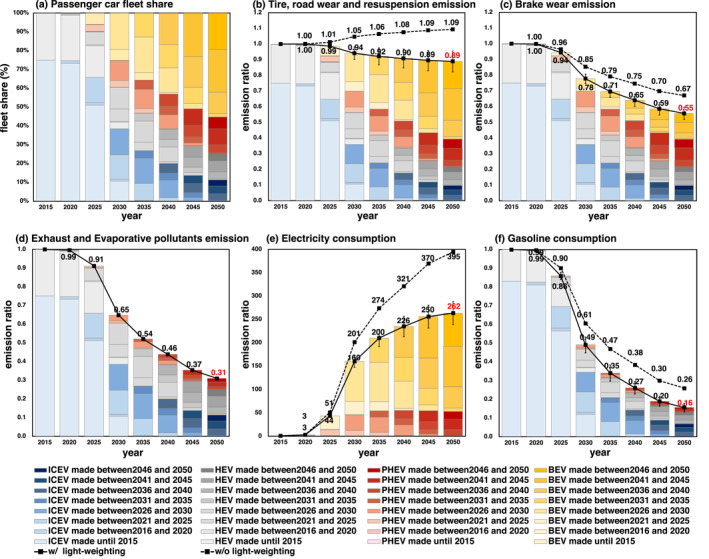
Trend in (a) passenger car fleet share, (b) tire and road wear and resuspension emission, (c) brake wear emission, (d) exhaust and evaporative pollutants emission, (e) electricity consumption, and (f) gasoline fuel consumption by passenger car through 2050. Each emission graph shows values relative to 2015. The bar graph and the solid black line indicate the trend with vehicle lightweighting. The dashed black line indicates the trend without vehicle lightweighting. The color shade indicate production year difference. The error bars indicate the uncertainty due to the degree of the technological progress of lightweighting (Figure 2). Since it is assumed that exhaust gas pollutants do not change with lightweighting (see Section 2.3.4.2), the error bars are not indicated in (d).

It is necessary to estimate the proportion of both vehicle type and production year in the passenger car fleet in 2050. This is because the vehicle production year affects fuel efficiency and vehicle weight. In this study, the vehicle turnover was estimated as a function of scrapping rate according to vehicle age, and the trend in the vehicle ownership share was predicted, as shown in Figure 3a. Figure S6 in Supporting Information S1 shows the vehicle type mix of new passenger car sales through 2050. This was estimated based on Sato and Nakata (2019) (based on data published by the METI, JAMA, and the Next‐Generation Vehicle Promotion Center (NGVP)). FCVs are expected to account for approximately 5% of the total sale share in 2050 (Sato & Nakata, 2019). Based on this, FCVs will not be major in terms of ownership share, so we excluded FCVs from this study. Figure S7 in Supporting Information S1 shows the scrap and residual rates as a function of vehicle age, derived by the Weibull functions shown in Equations 5 and 6. The Weibull function was first proposed by Weibull (1951), and it statistically represents the phenomenon of machine deterioration. It is widely used in the field of reliability engineering and has been used for modeling vehicle survivability (Hao et al., 2011).

(5) $$R\left(t\right)=\exp\left(-{\left(\frac{t}{\eta }\right)}^{m}\right),$$

(6) $$f\left(t\right)=\frac{m\cdot t^{m-1}}{\eta^{m}}\cdot \exp\left\{-{\left(\frac{t}{\eta }\right)}^{m}\right\},$$

where *R*(*t*) is the survival ratio at age *t* (years), and *f*(*t*) is the scrap ratio at age *t* (years). *η* is called scaling parameter and is defined as the average vehicle lifetime. Assuming *t* = *η* and substituting it into Equation 5, we obtain the residual *R*(*t*) = 1/*e*. *m* is called Weibull coefficient. In this study, *η* = 12.7 and *m* = 4.0 were used to reflect the residual pattern of ordinary vehicles in Japan (Lu et al., 2018). These values were derived by Lu et al. (2018) through regression against the patterns of residual rates derived from data on the numbers of registered and scrapped ordinary vehicles in Japan reported by Huo and Wang (2012). Figure 3a shows the vehicle type mix by production year for the total passenger car fleet in Japan through 2050. It was derived by assuming that cars are replenished by the share of new car sales in that year (Figure S6 in Supporting Information S1) for the number of scrapped cars in each year derived in Equation 5. The estimated vehicle ownership share in 2050 was 11% for ICEVs, 14% for HEVs, 20% for PHEVs, and 55% for BEVs (Figure 3a).

#### 2.3.4 Change in Emissions Through 2050

##### 2.3.4.1 Non‐Exhaust PM Emissions From Passenger Car

The emissions of “tire and road wear and resuspension” and “brake wear” in the 2050R&E&L experiment were estimated to be 89% and 55% of those in the BASE experiment, respectively (Figures 3b and 3c solid line). Figures 3b and 3c were derived by weighting the vehicle type mix by production year in Figure 3a by the vehicle weight in Figure 2 since both non‐exhaust PM emissions are proportional to vehicle weight (Simons, 2016). The brake wear was then further multiplied by 0.33 to take into account the effect of RBS for BEVs only. In the friction brake system (FBS) used in conventional vehicles, excess kinetic energy during braking is discarded as heat. In contrast, the RBS in BEVs can recover braking force by converting it into electrical energy. In BEVs, the combined use of the FBS and the RBS can reduce brake disc wear. Various values for the brake wear reduction effect of RBS have been reported in several references (Table 3). The value indicated by Hagino (2019), −67%, was adopted in this study. This is because PM emissions are directly measured by the Particle Measurement Program (PMP) test cycle, an expert working group for brake wear measurement, and non‐asbestos organic (NAO) brake pad material, which is the most common material used in Japan (accounts for approximately 70% share of passenger cars), is used. The brake wear reduction effect of the RBS was assumed to be −67%, even if the vehicle weight changed.

**Table 3:** Literature Values for Brake Dust Reduction Owing to the RBS of BEVs

Sector	Reported reduction ratio	Unchanged
Barlow (2014)	Almost −100%	Visual confirmation (Brake components look new after 22,000 miles driving)
Hooftman et al. (2016)	−66%	Replacement interval of brake pads (BEV's brake pad last approximately two‐thirds longer than that of diesel/petrol vehicles, in case of Tesla BMW i3 and LEAF)
Platform for Electro‐mobility (2016)	−25% to −50%	Information provided by company (Brake pad reduction ratio in case of Renault ZOE)
Kendrick and Kulkarni (2019)	−50%	Laboratory test (WLTP^a^ driving cycle and semi‐metallic brake pad)
Hagino (2019)	−67%	Laboratory test (PMP^b^ driving cycle and NAO^c^ brake pad)

*Note*. Much of this information is summarized in OECD (2020).

^a^ Worldwide‐harmonized light vehicles test procedure.

^b^ Particulate measurement program.

^c^ Non‐asbestos organic.

Primary emissions from tire and road wear and resuspension will be reduced to approximately 10% by around 2035 because of the body frame lightweighting of ICEVs and HEVs, but the reduction will stall after that (Figure 3b solid line) because BEVs will not be relatively lighter than ICEVs, HEVs, and PHEVs even in the future. As mentioned in Section 2.3.2, BEVs have a large rate of lightweighting and will be lighter than ICEVs produced in 2015 by 2030, so it was estimated that tire and road dust emissions will not increase in the future from the 2015 level.

In the case of the 2050R&E experiment, PHEVs are 8% heavier and BEVs are 14% heavier than ICEVs without considering future weight reductions (Figure 2). Therefore, the increase in the share of PHEVs and BEVs is estimated to increase tire and road wear and resuspension by 9% in 2050 relative to the reference experiment (Figure 3b dashed line). This is a disadvantage of BEVs, as mentioned in the Introduction. However, our estimation suggests that the disadvantages can be suppressed by lightweighting the body frame and battery (Figure 3b solid line).

Brake wear emissions will be reduced by 33% in 2050 compared to that in 2015 due to the penetration of BEVs even without considering vehicle lightweighting (Figure 3c dashed line) but can be reduced by 44% with lightweighting (Figure 3c solid line).

##### 2.3.4.2 Exhaust Gas and PM Emissions From Passenger Car

The exhaust gaseous pollutants emissions from passenger car will decrease as the fractions of BEVs and PHEVs increase. These in the 2050R&E and 2050R&E&L experiments were assumed to be 31% of those in the BASE experiment (Figure 3d). It was assumed that the emission factors of exhaust pollutants would not change even if fuel consumption was improved due to lightweighting. In addition, the emission factors for exhaust pollutants from gasoline driven HEVs and PHEVs were assumed to be the same as those for ICEVs. This is because while improved fuel consumption reduces CO_2_ emissions, but this is not true to trace pollutants such as NO_x_. HEVs are often driven by a motor using electricity generated by running the engine at low load and low speed. The low power operation of the engine may reduce exhaust emissions, but it may also increase emissions as the aftertreatment system takes longer to warm up and the catalyst stays at a lower temperature (Zhao and Wang, 2016).

For PHEVs, the ratio of electric driving to gasoline driving was assumed to be 7/3. The PHEV runs by externally charged electric power for a certain distance from the start of driving with a small drive battery of approximately 10 kWh and switches to engine‐driven hybrid running when the battery’s state of charge (SOC) decreases to a certain value. The ratio of electric drive to the total daily driving distance is called the utility factor (UF) (Society of Automotive Engineers J2841 standard). Since the UF varies by person and by day, previous studies have derived the average UF of PHEVs in Japan based on the statistical data of the daily distance traveled by PHEV users. It is estimated that the UF = 0.7 when assuming a PHEV with an electric driving range of 60 km, as represented by the Prius PHEV (Hori & Kaneda, 2012). Therefore, the electric driving ratio of PHEVs was assumed to be 70% in this study as well.

##### 2.3.4.3 Upper‐Stream Emissions (Thermal Power Plant and Gas Station)

The Japanese government expects an increase in renewable energy and a decrease in thermal power generation in the future in order to decarbonization. The emissions from power plants in the 2050 R&E&L experiment were assumed to be 18% of those obtained from the BASE experiment.

- The power supply mix in 2050 was estimated to be 50% renewable energy, 34% nuclear, and 16% thermal, provided that the government targets would be achieved (Figure S8b in Supporting Information S1).
- Thermal power generation, which provided 89% of the total electricity demand in 2012 (the PM2.5 EI base year) (Figure S8a in Supporting Information S1) (METI, 2019) would decrease by 82% by 2050.

The change in air pollutant emissions due to the introduction of CCUS (carbon capture, utilization and storage) in thermal power plants strongly depends on the type of CO_2_ capture technology employed (EEA, 2011) (Text S4 in Supporting Information S1). Furthermore, there are many uncertainties, including future innovations in denitrification and desulphurization technologies and regulatory changes, so estimating the changes in emission factors of thermal power plants is difficult. Therefore, the emission factors for pollutants from thermal power plants were assumed to remain the same in 2050 as in the base year.

No increase in power plant emissions was assumed for charging BEVs and PHEVs since it will be met by solar surplus electricity in 2050 (Text S5 in Supporting Information S1). In reality, surplus electricity can only be used for charging during the daytime. However, since the data of charging patterns for BEVs and PHEVs was not available, it was assumed that surplus electricity would be utilized without waste. In other words, it assumes enough stationary batteries to store solar surpluses. Figure 3e shows the electricity demand for external charging for passenger cars considering the improvement of fuel and electricity consumption (Table S6 in Supporting Information S1) due to lightweighting in 2050. The external charging electricity demand was negligible in 2015, as the share of BEVs and PHEVs in the total passenger car fleet was very small, approximately 0.2% (NGVP's website: https://www.cev-pc.or.jp/tokei/hanbaidaisu.html; Automobile Inspection & Registration Information Association (AIRIA)'s website: https://www.airia.or.jp/publish/statistics/trend.html, both in Japanese, last accessed: 22 January 2023). The additional electricity demand was only 0.02% of the total annual domestic electricity generation (107.78 billion kWh year^−1^; METI, 2019), assuming a total annual passenger car fleet of 420 billion km/year (MLIT, 2010) and an electricity consumption of 0.17 kWh km^−1^ for BEVs and PHEVs. In 2050, the demand for external charging electricity will increase by 260 times (Figure 3e solid line) due to the penetration of BEVs and PHEVs, which would increase the total electricity demand by 4% in Japan. However, it was estimated that this additional electricity demand could be met by surplus PV power (even in winter when solar radiation is low) (Figure S8b in Supporting Information S1). Without considering the improvement of electricity consumption due to lightweighting (2050R&E experiment), more electricity would be required for charging (395 times more than that in 2012 (Figure 3e dashed line), and 6% increase in the total demand). This may not be covered by some surplus power in the winter, but the increase in thermal generation would be approximately 1% at worst (Text S5 in Supporting Information S1).

The passenger car electrification and lightweighting will also reduce gasoline consumption. The decreasing refueling frequency will reduce fuel evaporation NMVOCs at gas stations. Figure 3f shows the gasoline demand by passenger cars. Gasoline consumption by passenger cars accounts for 80% of the total gasoline consumption (MLIT, 2012). With (without) vehicle lightweighting, it was estimated that electrification would reduce gasoline consumption in passenger cars by 84% (74%) (Figure 3f, solid (dashed) line) and NMVOCs evaporation from gas stations by 0.33 (0.41) times compared to those in the BASE experiment.

### 2.4 Observation Data for Model Validation

To validate the simulation results, nationwide seasonal observation data of Ministry of Environment (MOE), Japan, were used (available at http://www.env.go.jp/air/osen/pm/monitoring.html, last accessed: 25 January 2023)). The survey was conducted at 192 stations in Japan in 2015 (158 public, 44 roadside, and 15 background sites). During a period of 2 weeks × 4 seasons for a total of 56 days (once a day observation), daily concentrations of 32 elemental components (Cu, Fe, Mn, Ni, Pb, V, Zn, etc.), and 9 ionic components (NO_3_^−^, SO_4_^2−^, NH_4_^+^, Na^+^, K^+^, Mg^2+^, Cl^−^, Ca^2+^, and C_2_O_4_^2−^) were analyzed. The meteorological fields (temperature, pressure, wind speed, solar radiation, precipitation, and relative humidity) were also measured. The inorganic elemental components other than Si were mainly measured using inductively coupled plasma‐mass spectrometry (ICP‐MS) after acid decomposition with nitric acid, hydrofluoric acid, hydrogen peroxide, etc. The ionic components were analyzed using an ion chromatography. In this study, simulated values from four model grids adjacent to the observation point were weighted inversely proportional to the square of distances and used for comparison with the observations.

## 3 Result and Discussion

### 3.1 Model Evaluation

First, the reproducibility of metal concentrations is discussed. The scatter plots and comparative statistics of the simulation results and observations for PM_2.5_‐Fe, Cu, and Zn are shown in Figure S1 and Table S2 of the Supporting Information S1, respectively. The biases for Cu and Zn were significantly improved by the revision of the transition metal emission inventory TMI‐Asia/Japan from v1.0 to v1.1 in this study (Figure S1, Table S2 in Supporting Information S1). In v1.0, the normalized mean bias (NMB) of Cu ranged from 130% (roadside site) to 680% (background site) but improved to approximately 30% after the revision. In addition, the NMB of Zn at the background site improved from approximately 100% to −5% (Table S2 in Supporting Information S1). Therefore, the metal bias is within approximately 30% for Fe, Cu, and Zn (refer to Text S1 in Supporting Information S1 for details). The correlation coefficients are *R* = 0.37, 0.20, and 0.25 for Fe, Cu, and Zn, respectively. They were relatively high at the background site, *R* = 0.51, 0.35, and 0.50, respectively (Table S2 in Supporting Information S1), indicating that the model well reproduces the temporal concentration variations caused by advection from the continent. On the other hand, they were lower at the roadside sites, *R* = 0.28, 0.14, and 0.30. One possible cause is the dissociation between inventory and daily actual emissions. Therefore, the metal concentrations were discussed on a monthly or annual average basis in this study.

Next, PM_2.5_ concentrations and their ionic components in full chemistry simulations are described. The scatter plots and comparative statistics of the simulated and observed PM_2.5_ total mass concentrations and ionic components (SO_4_^2−^, NO_3_^−^, NH_4_^+^, Cl^−^, Na^+^, Ca^2+^, and Mg^2+^) are shown in Figure S9 and Table S7 of the Supporting Information S1, respectively. The model overestimated PM_2.5_ mass concentrations by approximately 60% throughout the year (Table S7 in Supporting Information S1). As for the main ionic components, the NMB of SO_4_^2−^ and NH_4_^+^ are −13% and −9%, respectively, but NO_3_^−^ is overestimated at 247% (Table S7 in Supporting Information S1). The overestimation of NO_3_^−^ is significant in summer (NMB = 94%, 199%, 2008%, and 234% in winter, spring, summer, and fall, respectively) (Table S7, Figure S9c in Supporting Information S1). Overestimation of simulated particulate nitrate in Japan has been a well‐known issue and reported for different regions, years, and models (e.g., Kajino et al., 2013; Shimadera et al., 2014). For example, Shimadera et al. (2014) conducted various sensitivity experiments using WRF‐CMAQ and showed that NH_3_ emissions and dry deposition may be particularly influential in model nitrate overestimation. However, the cause in this study is unclear. In this study, the concentrations of the sea salt particle components Cl^−^, Na^+^, and Mg^2+^ were also overestimated by a factor of 10 or more (Table S7, Figures S9e–S9h in Supporting Information S1), suggesting that the overproduction of NaNO_3_ due to chlorin loss (NaCl + HNO_3_ → NaNO_3_ + HCl) is another possible cause.

Also, the model also slightly underestimated the temperature (NMB = −10%) (Figure S10a in Supporting Information S1) and overestimated the relative humidity (RH) (NMB = 16%) (Figure S10b in Supporting Information S1). The uncertainties in strong acidic components NO_3_^−^ and RH affect the sensitivity of aerosol pH and thus metal solubility. In this study, the range of uncertainty in aerosol acidity was considered when calculating aerosol pH in ISORROPIA‐ll by also inputting the case corrected for bias from observations for the NO_3_^−^ concentration and RH, respectively. And the range of metal solubility due to NO_3_^−^ and RH uncertainties is described in Section 3.2.4.

### 3.2 Impacts of Renewable Energy Shifting, Passenger Car Electrification, and Lightweighting

#### 3.2.1 Impacts on Primary Emissions

##### 3.2.1.1 PM_2.5_ and PM_2.5_‐Metals

First, the metal content assumptions in PM_2.5_ in TMI‐Asia/Japan v1.1 are explained for estimating the primary emissions of metals. Power plants, automobile exhaust, brake and tire wear, and resuspension are shown in Table 4 (for other sectors, see Kayaba, 2023b). The fly ash from coal‐fired power plants contains approximately 1.0%–10% Fe, 0.01%–0.1% Cu, and 0.01%–1.0% Zn, respectively, in both PM_2.5_ and PM_10_ particle sizes (Chow et al., 2004). In this study, the assumptions for the Fe, Cu, and Zn content in thermal power plant exhaust PM_2.5_ were 4.2%, 0.07%, and 0.3%, respectively. The gasoline and diesel exhaust gases contain approximately 0.1%–1.0% Fe and Zn and 0.01%–0.1% Cu (Chow et al., 2004). Zn is included because zinc dithiophosphate is added to lubricants as an antiwear and antioxidant (Cadle et al., 1997; Lough et al., 2005). Fe and Cu are included mainly due to bearing wear and other component wear mixing (Cadle et al., 1997). The assumptions for the Fe, Cu, and Zn contents in vehicle exhaust PM_2.5_ in this study were 0.5%–0.7%, 0.03%–0.06%, and 0.2%–0.4%, respectively. The metal content in brake wear varies widely depending on the brake pad material. NAO, the most major brake pad material in Japan, contains almost no steel material. However, because of the cast iron component of the rotor (mating material), Fe is the most abundant metal in brake wear PM_2.5_ (Hagino, 2020; Hagino et al., 2016). The assumptions for Fe, Cu, and Zn contents in brake wear PM_2.5_ were 22%, 1.5%, and 1.3%, respectively, in this study. The composition of tire wear is mostly organic matter. Zn, added as a vulcanizing agent, is the most abundant heavy metal in tire wear, accounting for approximately 1% (Blok, 2005; Grigoratos & Martini, 2014; Smolders & Degryse, 2002). It is nearly impossible to separate primary road wear from other mineral dust deposited on roads (Denier van der Gon et al., 2013). Concrete and asphalt, the main components of road surfaces, are mineral aggregates comprising the crustal components Si, Ca, K, Fe, and Al, whose compositional ratios vary based on the geological source (Harrison et al., 2021). Resuspended particles consist of all non‐exhaust particles (brake, tire, and road wear) and particles from other sources deposited on the road surface (e.g., exhaust gas particles, particles from deicing and gritting, wind‐blown dust, and biogenic particles) (Harrison et al., 2021). The road dust sampling on asphalted roads in Portugal contained approximately 2%–5% PM_10_‐Fe and 0.03%–0.3% PM_10_‐Cu and PM_10_‐Zn (Alves et al., 2018). In this study, the assumptions for Fe, Cu, and Zn contents in road wear and resuspension PM were 3%, 0.03%, and 0.1%, respectively.

**Table 4:** Assumptions of the Metal Content Ratio in PM_2.5_ From Power Plant, Vehicle Exhaust, Brake Wear, Tire Wear, Road Wear and Resuspension Used in TMI‐Asia/Japan v1.1 Development

g‐metal/g‐PM_2.5_ in %	Fe	Cu	Zn
Thermal power plant	4.2	0.07	0.3
Vehicle exhaust	0.5–0.7^a^	0.03–0.06^a^	0.2–0.4^a^
Brake wear	22.2	1.5	1.3
Tire wear	0	0	1.0
Road wear and resuspension	3.0	0.03	0.1

^a^ The values used for different vehicle types (mini passenger car, passenger car, light duty truck, heavy‐duty truck, bus, and motorcycle) and subsectors by fuel (gasoline, diesel, and liquefied petroleum gas (LPG)) are indicated.

**Figure 4:**
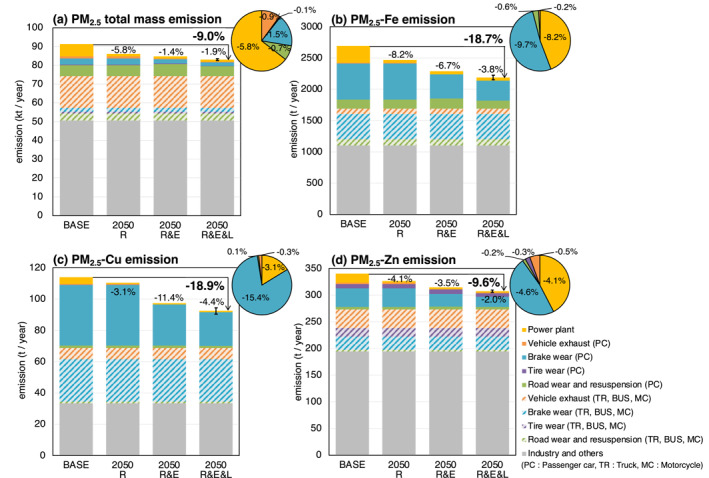
Total annual anthropogenic emissions of (a) PM_2.5_ total mass, (b) PM_2.5_‐Fe, (b) PM_2.5_‐Cu, and (c) PM_2.5_‐Zn in domain 02 Japan region. Comparison between the BASE experiment and each sensitivity experiment. The contributions of sectors to the reduction ratio in the 2050R&E&L case are indicated as a pie chart. The error bars indicate the uncertainty due to the degree of the technological progress of lightweighting; high and low (Figure 2). The change ratio shown for each sensitivity experiment represent is based on the BASE experiment. “Industry and others” sector include aviation, navigation, railway, domestic, cooking, incineration, and field burning.

Figure 4 shows the total annual emissions of the anthropogenic PM_2.5_ total mass, Fe, Cu, and Zn in domain 02 Japan region and reductions by 2050 due to the renewable energy shifting, vehicle electrification, and lightweighting. To summarize, in the 2050R&E&L experiment, Fe and Cu were estimated to be reduced by approximately 19%, Zn by 10%, and PM_2.5_ total mass by 9%. The decisive factor in the difference in these reduction rates is the difference in the brake wear‐derived contribution to total emissions. In the 2050R&E&L experiment, brake wear has the largest reduction rate in emission factors than tire and road wear and resuspension due to the double effect of the RBS and lightweighting of BEVs. Therefore, the emissions of Fe and Cu, which highly depend on brake wear, were most significantly reduced.

For the reductions in primary PM_2.5_ total mass emissions, renewable energy shifting contributed the most (−5.8%) (Figure 4a). In the case of passenger car electrification without lightweighting (2050R&E−2050R), the decrease in PM_2.5_ from brake wear and exhaust gas would be partially offset by the increase in PM_2.5_ from tire wear, road wear, and resuspension. As a result, the net reduction in PM_2.5_ emissions was −1.4%, although it did not increase. Lightweighting prevents the increase in tire wear, road wear, and resuspension, further reducing PM_2.5_ emissions by 1.9%. However, passenger car exhaust/non‐emission PM_2.5_ accounts for approximately only 10% of total emissions, and in any case, the PM_2.5_ reduction effect of electrification and lightweighting is limited (−3.2%). The largest sources of Fe emissions in Japan are brake wear and the steel industry (included in “Industry and others”). When PM_2.5_ from brake wear is reduced by 45% by passenger car electrification and lightweighting (Table 1), it contributes to a 9.7% reduction in total Fe emissions. When PM_2.5_ from thermal power plants is reduced by 82% by renewable energy shifting (Table 1), it contributes to an 8.2% reduction in total Fe emissions. As a result, reductions in brake wear and thermal power plants contributed roughly equal to the reduction in Fe emissions (−18.7% ± 1.4%) in 2050 (Figure 4b). The largest emission source of Cu is brake wear, accounting for 60% of total emissions. Therefore, the reduction in Cu emissions in 2050R&E&L (−18.9% ± 1.8%) is mostly due to the reduced brake wear (−15.4%) due to passenger car electrification and lightweighting (Figure 4c). Zn is characterized by having a tire wear‐derived source. In the case of passenger car electrification without lightweighting (2050R&E−2050R), Zn emissions increase from tire and road wear and resuspension (+0.3%) but decrease more from brake wear (−4.0%), resulting in a net decrease (−3.5%) (Figure 4d). The error bars in Figure 4 show the range of lightweight technology progress. The uncertainties are estimated to be ±0.6%, ±1.4%, ±1.8%, and ±0.7% of the PM_2.5_ total mass, Fe, Cu, and Zn emissions in the 2050 R&E&L experiment, respectively, which are relatively small. Therefore, hereafter, the lightweight technology progress is discussed in terms of low and high averages.

##### 3.2.1.2 Gaseous Pollutants

**Figure 5:**
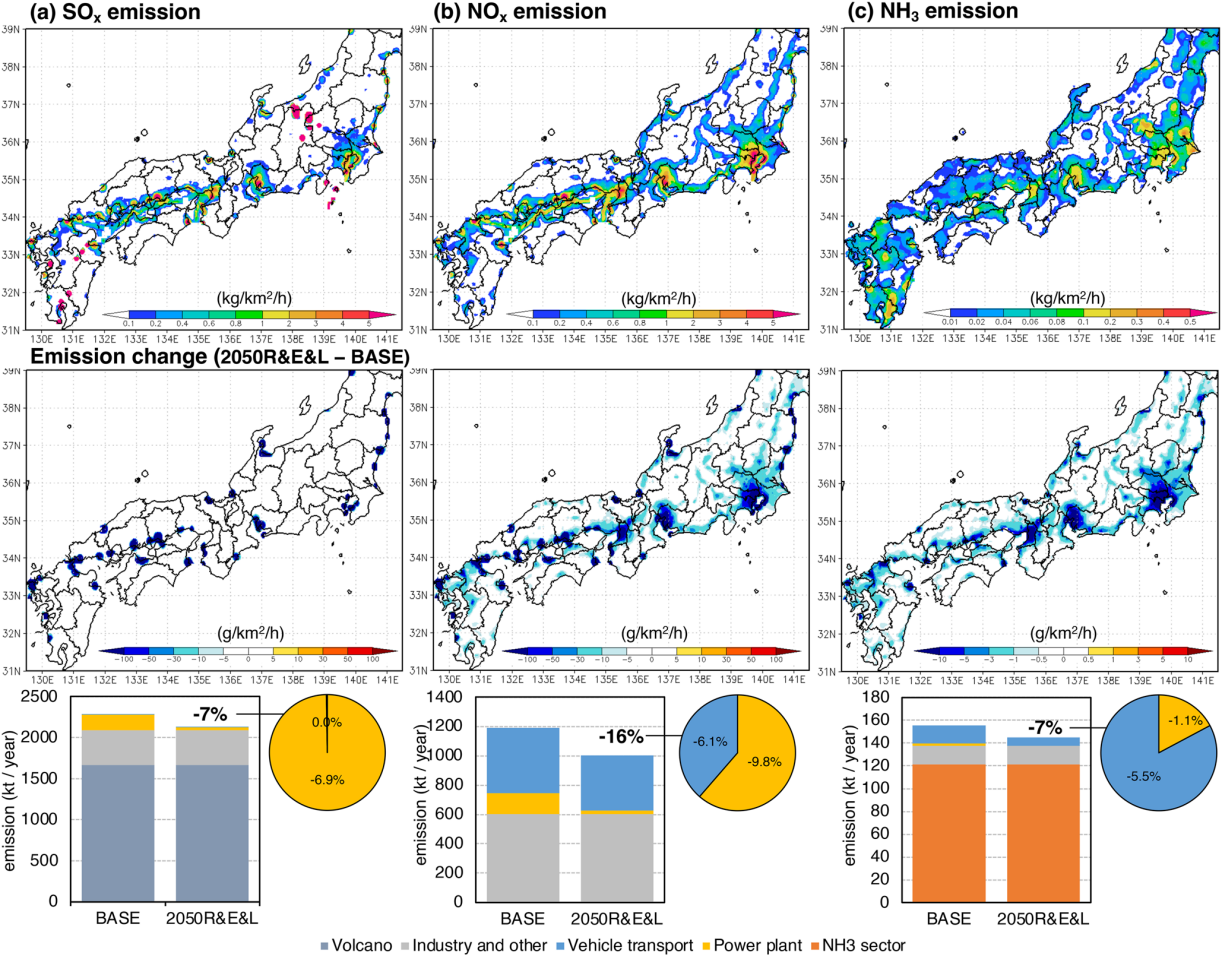
Total annual emissions of (a) SO_x_, (b) NO_x_, and (c) NH_3_ in domain 02 Japan region (top), and emission change in the 2050R&E&L experiment (middle). The bar graphs show the breakdown of the total domestic emission in the BASE experiment and the 2050R&E&L experiment. The contribution of the sectors to the reduction rate is shown as a pie chart. “Industry and other” includes aviation, navigation, domestic, cooking, incineration and field burning. “NH_3_ sector” includes livestock, agriculture, and drainage.

Figure 5 shows the primary emissions of SO_x_, NO_x_, and NH_3_ in domain 02 Japan region. In the 2050R&E&L experiment, they were reduced by 7%, 16%, and 7%, respectively, in comparison to the base experiment. The SO_x_ emissions reduction is almost entirely due to the reduction in thermal power generation (Figure 5a). The limit of sulfur content in fuel is lower than 0.001% in Japan (CEC, 2003) to maintain the performance of diesel particulate filters (DPFs), so there is originally almost no emission from vehicles. Both passenger cars and thermal power plants contribute to the reduction of NO_x_ emissions, while the latter contributes more (−9.8%). The domestic NO_x_ emissions would only be reduced by −6.1% (Figure 5b), even in the case of a 70% emission reduction due to the passenger car electrification in 2050 (Table 1), because of the high contribution of heavy‐duty diesel vehicles. NH_3_ emissions reduction was mainly brought by vehicles. NH_3_ is generated in power plants during the denitration process and from vehicles as a byproduct of selective catalytic reduction in diesel vehicles and three‐way catalysts in gasoline vehicles. The change in energy and vehicles contributed to 1.1% and 5.5% NH_3_ emissions reduction in 2050, respectively (Figure 5c). NMVOCs are precursors of O_3_ and contribute to the formation of secondary aerosols. NMVOC emissions decreased by 2% in July and 7% in December due to lower vehicle exhaust emissions and fuel evaporation at fueling stations (figure omitted).

#### 3.2.2 Impacts on Atmospheric PM_2.5_ and PM_2.5_‐Metals Concentration

**Figure 6:**
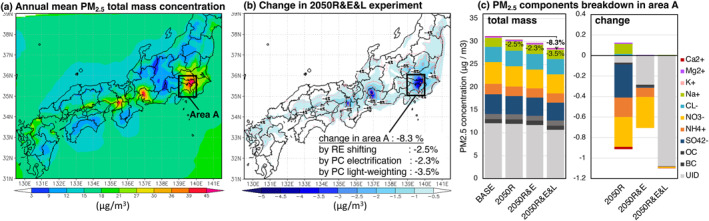
(a) Annual mean concentration of PM_2.5_ total mass in the BASE experiment and (b) change in the 2050R&E&L experiment. The change in area *A* and that due to renewable energy (RE) shifting, passenger car (PC) electrification, and PC lightweighting alone are shown in (b). (c) Shows the breakdown of PM_2.5_ components in area *A* (139–140°E, 35–36°N; urban area including Tokyo).

Figure 6 shows the change in PM_2.5_ total mass concentration. In the 2050R&E&L experiment, the PM_2.5_ concentration was reduced by 8.3% in area A (Figures 6a and 6b).

In area *A*, renewable energy shifting (2050R–BASE) reduced secondary‐formed PM_2.5_ (nitrate and sulfate) more than primary emission‐derived PM_2.5_ (i.e., UID, BC, and OC) (Figure 6c). The reduction of SO_x_ from the thermal power plant freed cations (NH_4_^+^, Na^+^, and Mg^2+^) in sulfate, which reacted with HNO_3_ to form nitrate, thus increasing Na^+^ (i.e., the reduction of NO_3_^−^ was probably partially offset). The electrification of passenger cars (2050R&E–2050R) reduced exhaust‐derived nitrate and non‐exhaust‐derived UID by about the same amount (Figure 6c). While the reduction of the PM_2.5_ concentration by the electrification of passenger cars alone is −2.3%, the reduction in non‐exhaust PM due to lightweighting (2050R&E&L–2050R&E) increased the effect by more than twice (−5.8%).

**Figure 7:**
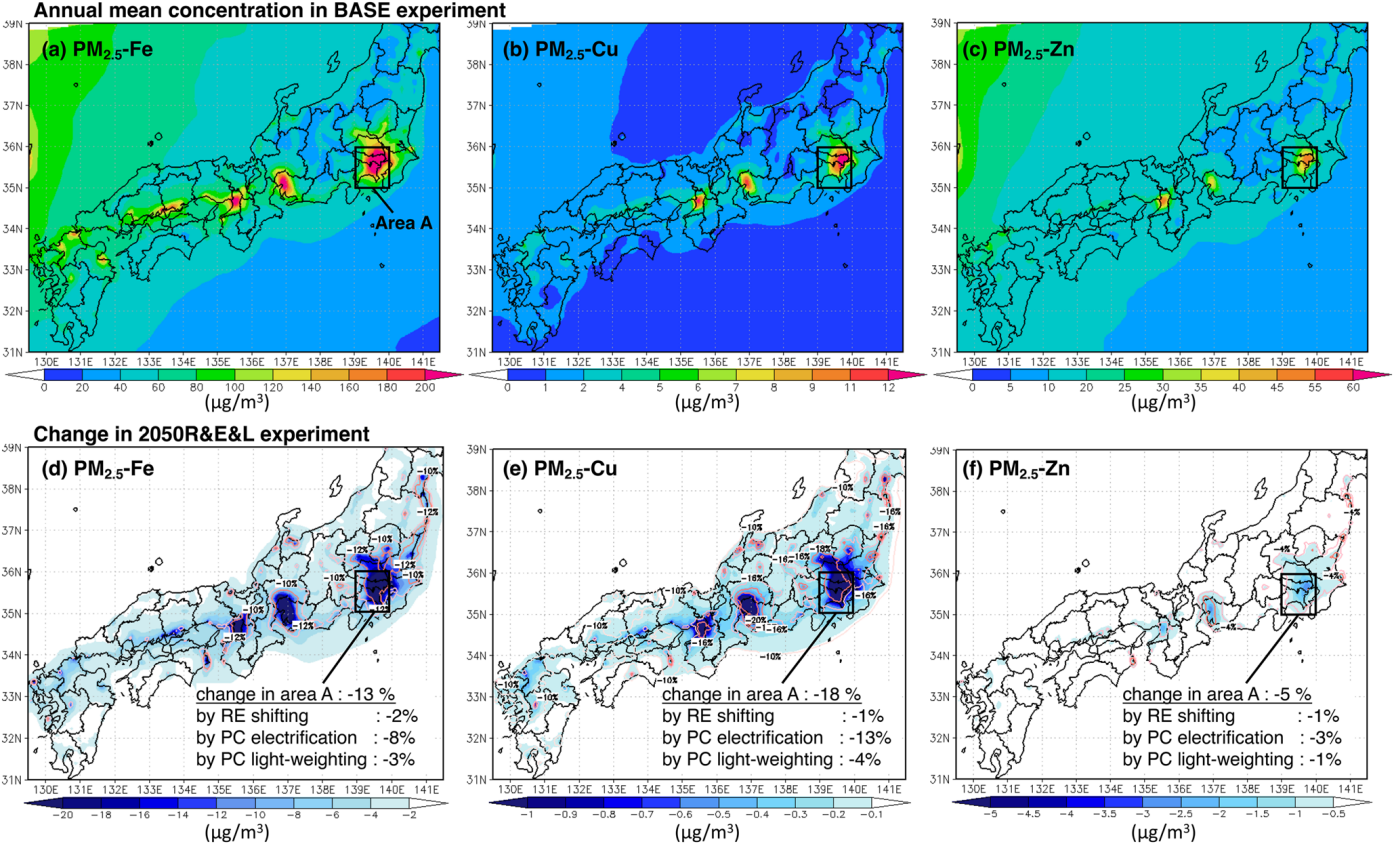
Annual mean concentrations of (a) PM_2.5_‐Fe, (b) PM_2.5_‐Cu, and (c) PM_2.5_‐Zn in the BASE experiment. (d–f) Are the change in (a)–(c) in the 2050R&E&L experiment. The change in area A and that due to renewable energy (RE) shifting, passenger car (PC) electrification, and PC lightweighting alone are shown in the figure (d–f). As in Figure 6, the urban area is enclosed as area *A* (139–140°E, 35–36°N).

Figures 7a–7c shows the annual mean concentrations of Fe, Cu, and Zn in PM_2.5_ in the BASE experiment. The concentrations are high in urban areas in the Kanto, Kansai, and Chubu regions, which are major emission sources. For Fe and Zn, the concentration gradient between the Japan Sea and the Japanese Islands is more gradual than that for Cu, suggesting a relatively higher contribution from continental advection. As a result of source–receptor analysis, the contribution of the emissions from the Asian continent was high for Zn, Fe, and Cu in that order, and the seasonal variations associated with continental advection (high concentrations in winter and spring, low concentrations in summer) were distinct in the same order (Figure S11 in Supporting Information S1). The effect of reducing domestic emissions was higher in the summer months when the continental contribution was lower for all metals.

In the 2050R&E&L experiment, Fe, Cu, and Zn concentrations were reduced by 13%, 18%, and 5%, respectively, in area *A* (Figures 7d–7f). The rate of primary emission reduction for Fe and Cu was similar at −19% (Figures 4b and 4c), but Cu reduced the concentration more. This may be because brake wear was the dominant contributor to the primary emission reduction of Cu, effectively reducing the areal concentration. For Fe, the contribution of power plants to emission reductions was higher than for Cu, indicated by more localized reductions in concentrations near coastal thermal power plants in East Japan (Figure 7d). To summarize, Cu and Fe had relatively high concentration reductions. This is because they had a large contribution of brake wear‐derived and reflected the significant benefit of RBS due to the penetration of BEVs. On the other hand, PM_2.5_ total mass and Zn had relatively small reduction rates due to the high contribution of industry and heavy‐duty vehicle exhaust (unchanged in this sensitivity experiment).

#### 3.2.3 Impacts on Aerosol Acidity

**Figure 8:**
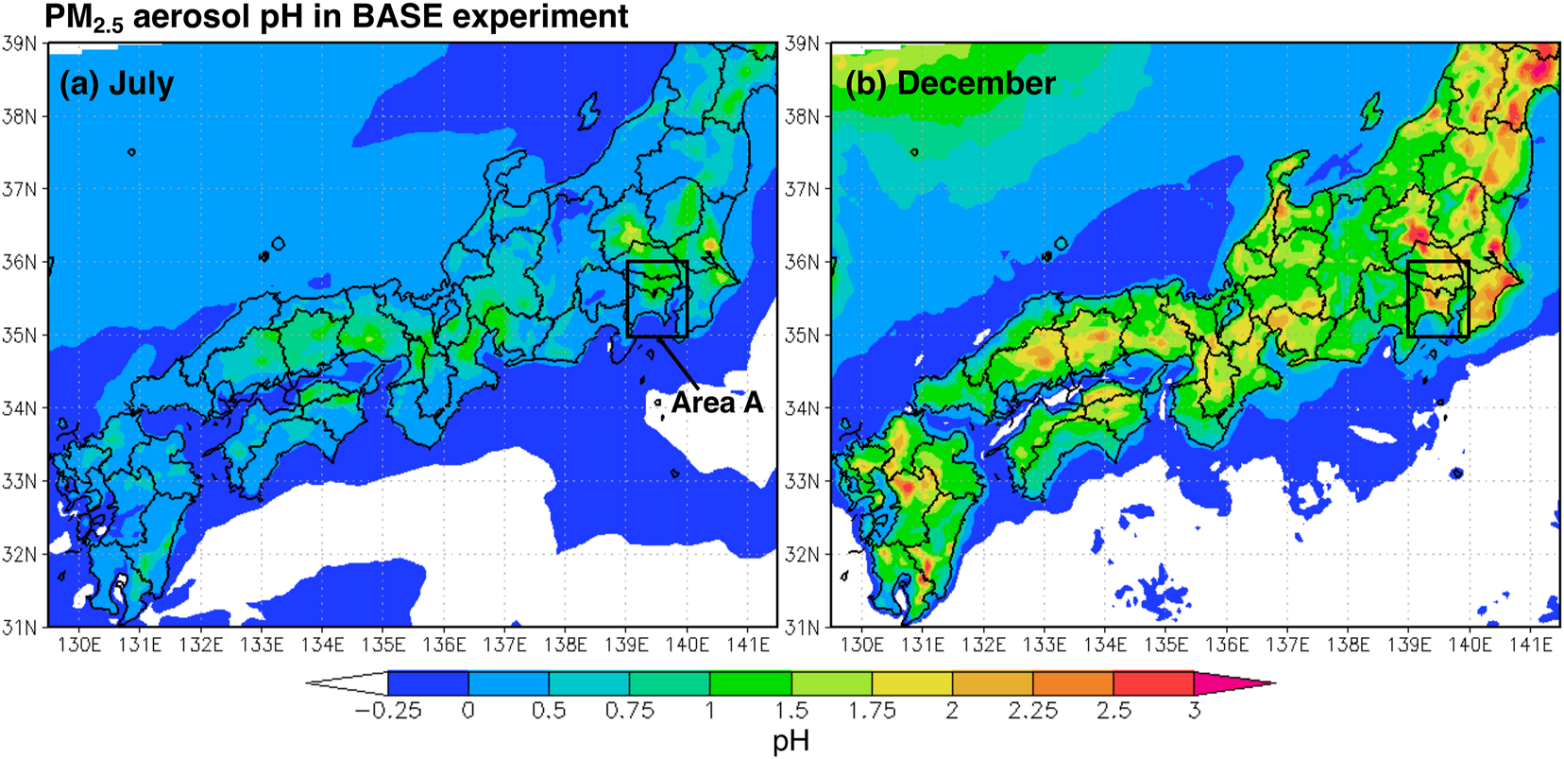
Simulated monthly mean of PM_2.5_ aerosol pH in (a) July and (b) December in the BASE experiment, averaged using only hourly data of 20% < RH < 95%. As in Figure 6, the urban area is enclosed as area *A* (139–140°E, 35–36°N).

Figure 8 shows the monthly mean aerosol pH in July and December. July and December were chosen because pH is generally lower in summer and higher in winter. The main reason for the low pH during the summer months is the high concentration of oxidants due to high solar radiation, which promotes sulfate formation (Guo et al., 2018; Song & Osada, 2020). NH_4_^+^ and NO_3_^−^ migrate to the gas phase at high temperatures, while SO_4_^2−^ is almost always in the particulate phase due to its low vapor pressure. The loss of NH_4_^+^ from NH_4_NO_3_ and (NH_4_)_2_SO_4_ predominates over the loss of NO_3_^−^ only from NH_4_NO_3_, and the net increase in particle H^+^ reduces pH (Guo et al., 2018). The pH was estimated to range from 0.5 to 1.5 in July and from 1 to 3 in December in this study.

**Figure 9:**
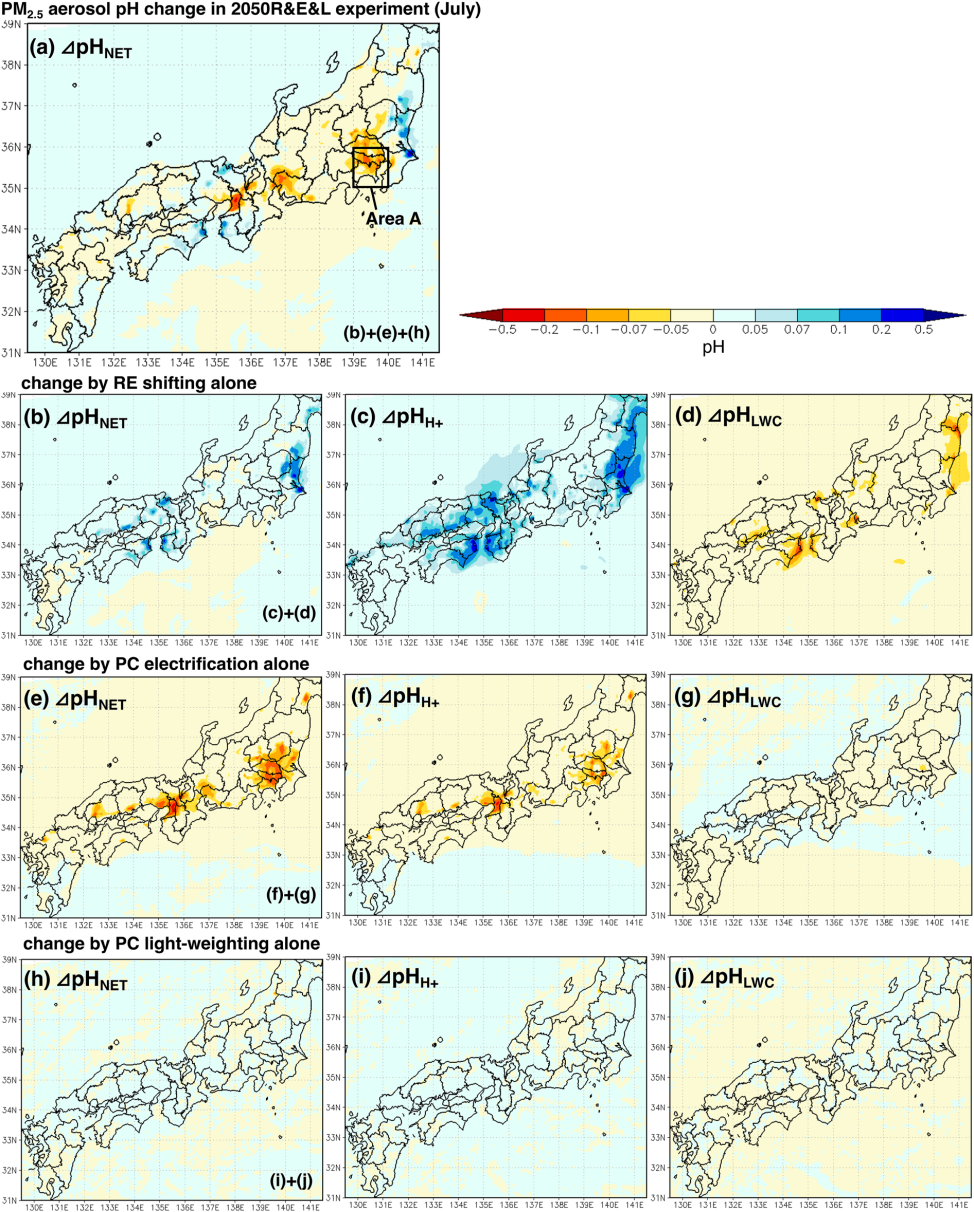
(a) PM_2.5_ aerosol pH change in the R&E&L experiment in July. Rows 2, 3, and 4 are the aerosol pH change by (b) renewable energy shifting, (e) passenger car (PC) electrification, and (h) PC lightweighting alone, respectively. The sum of $\Delta {\text{pH}}_{H^{+}}$ (center) and the ΔpH_LWC_ (right) is ΔpH_NET_ (left). The $\Delta {\text{pH}}_{H^{+}}$ is the effect of the H^+^ process, that is change in the amount of hydronium ions due to change in acidic or basic substances (c, f, i). The ΔpH_LWC_ is the effect of the LWC process, which is the change in aerosol water content due to the change in the mass of water‐soluble aerosols (d, g, and j). As in Figure 6, the urban area is enclosed as area *A* (139–140°E, 35–36°N).

Figure 9a shows the change in aerosol pH in July in the R&E&L experiment. Figures 9b, 9e, and 9h show the sensitivity by renewable energy shifting, passenger car electrification and lightweighting alone, respectively. The results for December are also shown in Figure S17 of the Supporting Information S1. The renewable energy shifting decreased aerosol acidity (maximum pH +0.2) in areas near power plants (Figure 9b), and the passenger car electrification increased aerosol acidity (maximum pH −0.2) in urban areas (Figure 9e). The aerosol acidity increased slightly net in urban areas in 2050 (maximum pH −0.1) (Figure 9a).

The explanation of the pH change due to renewable energy shifting is relatively simple. Renewable energy shifting mainly reduces SO_x_ and NO_x_ emissions from power plants but has a small effect on NH_3_ reduction. Also, because SO_4_^2−^ is nonvolatile, the effect on pH due to the SO_x_ emission control is not affected by gas–aerosol distribution, contrary to the case of NO_x_ and NH_3_ emission control. Therefore, $\Delta {\text{pH}}_{H^{+}}$ increased (Figure 9c). Although the LWC decreases as SO_4_^2−^ decreases, the freed cations (such as NH_4_^+^ and Na^+^) form nitrate with HNO_3_ (Seinfeld & Pandis, 2016), which may partially offset the LWC decrease. Despite the reduction in power plant NO_x_ emissions, increases in nitrate partially occurred in this study as well (Figure S16 in Supporting Information S1), but the effect of sulfate reduction was larger, resulting in a net decrease in LWC (ΔpH_LWC_ decrease) (Figure 9d). Finally, the effect of H^+^ reduction (pH increase) (Figure 9c) was greater than the effect of concentration by LWC reduction (pH decrease) (Figure 9d), resulting in a net pH increase (Figure 9b).

The vehicle electrification contributed to the pH decrease (Figure 9e). The reduction of on‐road NO_x_ and NH_3_ resulted in an increase in H^+^ ($\Delta {\text{pH}}_{H^{+}}$ decreased) in urban areas in July as a result of the acid–base balance (Figure 9f). However, in December, unlike the trend in July, the distribution of $\Delta {\text{pH}}_{H^{+}}$ was random with mixed positive and negative values and offset each other (Figure S17f in Supporting Information S1). These differences in pH change are due to seasonal differences in aerosol pH. The aerosol pH was lower in summer, and NH_4_^+^ tended to be more present in the particle phase than NO_3_^−^, so NH_3_ emission reduction was effective in reducing NH_4_^+^ (i.e., increasing H^+^) in the particle phase. This mechanism of seasonal differences in aerosol pH change due to NO_x_ and NH_3_ emission controls is discussed in detail in Appendix A. The effect of ΔpH_LWC_ was small in both summer and winter (Figure 9g).

The vehicle lightweighting had little effect on either the H^+^ or LWC process (Figures 9i and 9j). Note that the impact of light‐weighting alone is due only to reduced fuel evaporative NMVOCs emissions at the gas station and not to any change in on‐road emissions (Table 1).

#### 3.2.4 Impacts on Water‐Soluble Metals

Finally, the change of water‐soluble metal concentrations is discussed, considering the water solubility of metals depending on aerosol pH. This was analyzed using the relationship between metal solubility and aerosol pH by Fang et al. (2017) and Baldo et al. (2022) (Figure S18 in Supporting Information S1). The solubility of metals increases significantly below pH 2–3 (Fang et al., 2017; Wong et al., 2020).

The change in metal solubility due to emission changes in 2050 (2050R&E&L‐BASE) was smaller than that due to changes in total metal concentrations (i.e., changes in primary metal emissions). Note that the uncertainties in the NO_3_^−^ concentration and RH of the model simulations may result in biases less than ±1% and ±3% for changes in the water‐soluble fractions of Fe and Cu, respectively. In addition, water‐soluble fractions may be affected by history in past transport pathways (Wong et al., 2020), but that effect was not considered in this study. In addition, the solubility of metals is promoted by not only changes in aerosol pH (i.e., proton‐driven) but also by complex formation with organic matter and their photoinduced dissolution. Laboratory study has reported that the solubility of Fe at pH 2 by oxalates under dark conditions was four times greater than that for proton‐promoted dissolution, and photoinduced dissolution was twice greater than that found under dark conditions (Chen & Grassian, 2013; Ito, 2015). The effects of such ligand‐drive and photoinduced dissolution were not considered in this analysis. Compared to the ranges of these uncertainties and the seasonal variation of aerosol pH, the change in metal solubility in the 2050R&E&L experiment was estimated to be very small.

Table 5 summarizes the change of total metal concentrations, water‐soluble fractions of metals, and water‐soluble metal concentrations in area *A*. Their respective spatial distributions are shown in Figure S19 of the Supporting Information S1. The change of water‐soluble metal concentrations mainly depends on (1) changes in primary metal emissions and less on (2) changes in aerosol acidity. Therefore, the primary emission control of metals is more important than gaseous pollutants in reducing water‐soluble metal concentrations.

**Table 5:** The Change of Total Metal Concentration, Water Soluble Fraction of Metals and Water‐Soluble Metal Concentration in Area *A* (139–140°E, 35–36°N)

		Total metal concentration (ng/m^3^)		Water‐soluble fraction of metals (%)		Water‐soluble metal concentration change (%)		
		BASE	2050R&E&L	BASE	2050R&E&L	(1) by metal primary emission change	(2) by aerosol acidity change	NET
July	Fe	132	113	39^a^	40^a^	−14.6	+1.4	−13.2
	Fe	132	113	37^b^	38^b^	−14.6	+2.4	−12.2
	Cu	6	5	100^a^	100^a^	−18.6	±0.0	−18.6
	Zn	20	19	100^c^	100^c^	−6.3	±0.0	−6.3
December	Fe	169	149	23^a^	23^a^	−12.2	+0.1	−12.0
	Fe	169	149	11^b^	11^b^	−12.2	+0.4	−11.7
	Cu	7	6	52^a^	51^a^	−17.7	+1.5	−16.2
	Zn	30	29	52^c^	51^c^	−4.5	+1.7	−2.8

^a^ Derived from the relationship between aerosol pH and water‐soluble fraction of metals indicated by Fang et al. (2017) (Figure S18 in Supporting Information S1).

^b^ Derived from the relationship between aerosol pH and water‐soluble fraction of metals indicated by Baldo et al. (2022) (Figure S18 in Supporting Information S1).

^c^ Since there is no information on the water‐soluble fraction of Zn, it was assumed to be the same as that of Cu.

## 4 Conclusions and Future Issue

The impacts of renewable energy shifting, passenger car electrification, and lightweighting through 2050 on the atmospheric concentrations of PM_2.5_ total mass and oxidative stress‐inducing metals (PM_2.5_‐Fe, Cu, and Zn) in Japan were evaluated using a regional meteorology–chemistry model. The domestic primary emissions of PM_2.5_ total mass, Fe, Cu, and Zn reduced by 9%, 19%, 18%, and 10%, and their surface concentrations in the urban area decreased by 8%, 13%, 18%, and 5%, respectively.

BEVs have been considered to have no advantage in terms of non‐exhaust PM emissions by previous studies. This is because the disadvantages (heavier weight increases tire wear, road wear, and resuspension) offset the advantages (RBS reduces brake wear). However, the future lightweighting of drive battery and body frame were estimated to reduce all non‐exhaust PM. Passenger car electrification alone only reduced PM_2.5_ concentration by 2%. However, Fe and Cu concentrations were more reduced (−8% and −13%, respectively) because they have high brake wear‐derived and significantly reflects the benefits of BEV's RBS.

The water‐soluble metal concentrations (induce oxidative stress in the body) were estimated based on aerosol acidity. The renewable energy shifting mainly reduced SO_x_ and NO_x_ from thermal power plants, and the passenger car electrification mainly reduced tailpipe‐derived NO_x_ and NH_3_, which slightly changed aerosol acidity in urban areas (max pH ±0.2). Even if passenger car electrification reduces NO_x_ and NH_3_ simultaneously, the NH_3_ reduction effect might be dominant, that is, aerosol acidity might increase. This is because NH_4_^+^ tends to be more present in the particle phase than NO_3_^−^ in the summer when the ambient aerosol pH is low. However, anyway, the change in aerosol acidity had negligible effect on water‐soluble metal concentrations (maximum +2% for Fe, +0.5% for Cu, and Zn).

Therefore, the metal emissions reduction was more important than gaseous pollutants in decreasing the water‐soluble metals that induce respiratory oxidative stress and passenger car electrification and lightweighting were effective means of achieving this.

Finally, we present recommendations for future modeling studies to predict the risk of respiratory oxidative stress due to air pollutants.

1. Consideration of PAHs and PAH quinones in the model. PAH quinones, as well as transition metals, catalyze ROS production through the redox cycle (Charrier & Anastasio, 2012; Jiang et al., 2019; Kumagai et al., 2002; McWhinney et al., 2013). In addition, even if exposed as PAHs, they are converted to PAH quinones in the body by reductases such as cytochrome P‐450 (Hrdina et al., 2022; Jiang et al., 2019; Kumagai et al., 2012). Also, it is necessary to analyze considering future scenarios for heavy‐duty vehicles, which are one of the major sources of PAH.
2. Consideration of the source‐dependent solubility of metals. The Fe solubility of pyrogenic aerosols such as biomass burning and fossil fuel combustion varies greatly depending on the source and can be one to two orders of magnitude higher than that of lithogenic aerosols (as low as 0.5%) (Ito et al., 2021). Oakes et al. (2012) estimated Fe solubility in automobile exhaust and biomass burning to be 51%–75% and 46%, respectively. The solubility of aerosol Fe in coal fly ash (present as glassy Fe (oxyhydroxide aggregates)) was reported to be less than 1%, while the that of oil fly ash (present as ferric sulfate salt) was as high as 36% (Desboeufs et al., 2005) and even approximately 80% (Schroth et al., 2009). In metal modeling, it is ideal to be able to set the initial solubility rate at primary emissions linked to emission inventories, in addition to the atmospheric process of changing solubility due to protons and ligands.
3. Consideration of organic matter in the model. The interaction of organics (such as HULIS and PAH quinones) and transition metals have synergistic, additive, or antagonistic effects on OPDTT (Lin & Yu, 2020; Xiong et al., 2017; Yu et al., 2018). Water‐soluble organic compounds contain atmospheric ROS (H_2_O_2_, ROOH), which decompose in the body to bring OH radicals (Tong et al., 2016). Furthermore, the complex formation of metals with the organic ligands of oxalates solubilizes the metals (Chen & Grassian, 2013; Wong et al., 2020; Zhou et al., 2015). Therefore, it is important to consider the organic matter in terms of its own ROS‐producing capacity and metal solubilization.
4. Consideration of differences in metal solubility between the atmosphere and the body. Because the respiratory tract is water‐saturated and the alveolar epithelial lining fluid is weakly basic, the solubility of metals may be different in the atmosphere and the body.
5. Improvement of the reproducibility of nitrate concentration by NHM‐Chem. The current NHM‐Chem overestimated NO_3_^−^ especially in the summer. This overestimation did not substantially affect the main results of this study, but should be resolved in the future using size‐resolved measurements of inorganic compounds.
6. Assessment of respiratory oxidative stress risk. This study evaluated the impacts of future energy and vehicle transitions on atmospheric concentrations of water‐soluble metals. In the future, it would be desirable to estimate the changes in the risk of respiratory oxidative stress using, for example, a dose‐response assessment.

## Conflict of Interest

The authors declare no conflicts of interest relevant to this study.

## Supporting information

Supporting Information S1

## Figures and Tables

**Figure A1:**
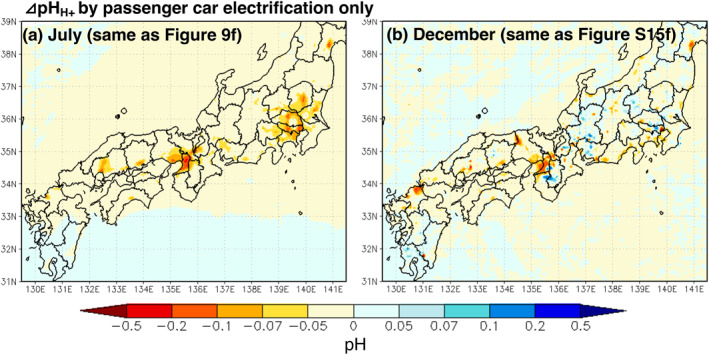
Change in $\Delta {\text{pH}}_{H^{+}}$ due to passenger car electrification only. Same as Figure 9f and Figure S15f in Supporting Information S1 for (a) July and (b) December, respectively.

**Figure A2:**
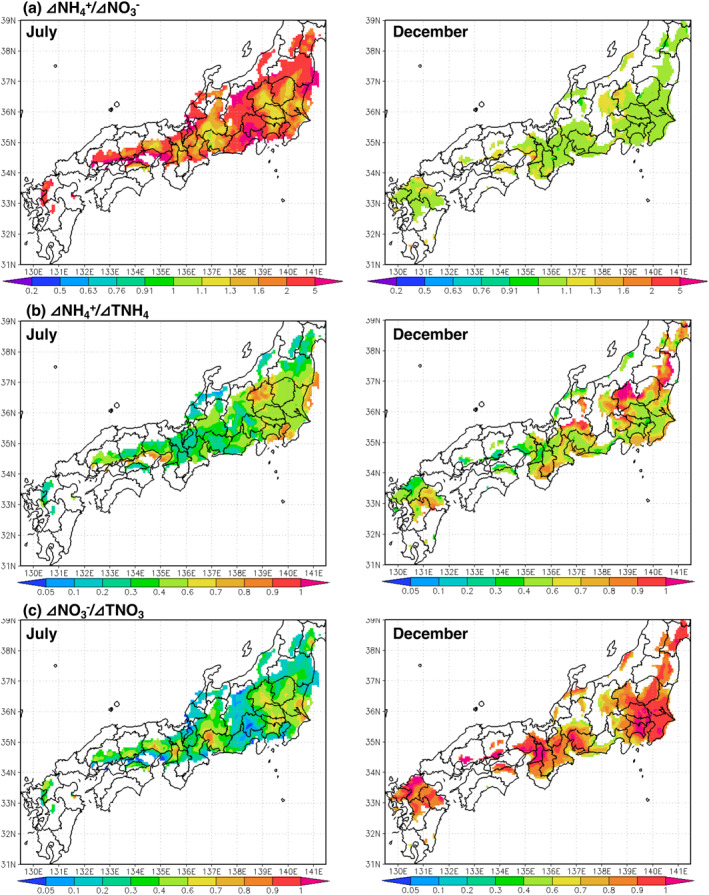
(a) Reduction ratios of NH_4_^+^ and NO_3_^−^ concentrations (⊿NH_4_^+^/⊿NO_3_^−^) by passenger car electrification only (2050R&E–2050R), (b) ratio of NO_3_^−^ in the reduction of TNO_3_ (=NO_3_^−^ + HNO_3_), and (c) ratio of NH_4_^+^ in the reduction of TNH_4_ (=NH_4_^+^ + NH_3_). Only the grids with TNO_3_ and TNH_4_ reduction ratios greater than 5% were drawn.

**Figure A3:**
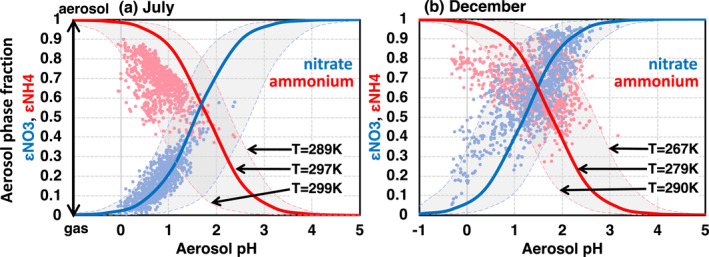
Relationship of $\varepsilon {\text{NO}}_{3}^{-}$ and $\varepsilon {\text{NH}}_{4}^{+}$ to ambient aerosol pH in (a) July and (b) December. The scatter plots are the results of the model simulation (2050R experiment). Only data from grids that were relatively sensitive ($\Delta {\text{pH}}_{H^{+}}$ > 0.05) in Figures A1a and A1b are plotted for the purpose of interpreting their positive and negative sensitivity. The S‐curves were derived using the theoretical equation proposed by Guo et al. (2017) shown in Equations A1 and A2, with monthly averages of temperature and LWC for the model grid of interest (July: *T* = 297 K, *C*_*w*_ = 12.7 μg m^−3^; December: *T* = 279 K, *C*_*w*_ = 5.0 μg m^−3^). The gray shading indicates the max–min temperature range and LWC. These figures were drawn using the spread sheet provided as Supporting information of Nenes et al. (2020).

**Figure A4:**
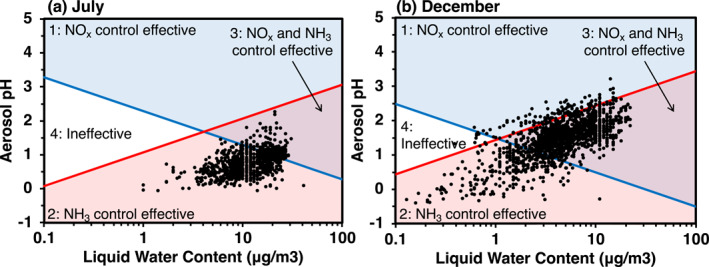
Evaluation of chemical sensitivity windows of the particle phase by NO_x_ and NH_3_ emission control. The effectiveness of NO_x_ and NH_3_ emission control is classified into four “windows” depending on the LWC and aerosol pH conditions. The solid blue (or red) line is the boundary of $\varepsilon {\text{NO}}_{3}^{-}$ (or $\varepsilon {\text{NH}}_{4}^{+}$) = 0.3 derived by the theoretical equation of Guo et al. (2017) shown in Equations A1 and A2. In the windows where pH is below the blue line or above the red line, NO_3_ and NH_4_ are relatively abundant in the gas phase (>70%) and NO_x_ (NH_3_) emission control is considered ineffective. The temperatures are the monthly average values (July: *T* = 297 K, December: *T* = 279 K). The plots are model simulation result data for the grid with $\Delta {\text{pH}}_{H^{+}}$ > 0.05 in Figures A1 and A3. These figures were drawn using the spread sheet provided as Supporting Information of Nenes et al. (2020).

**Figure A5:**
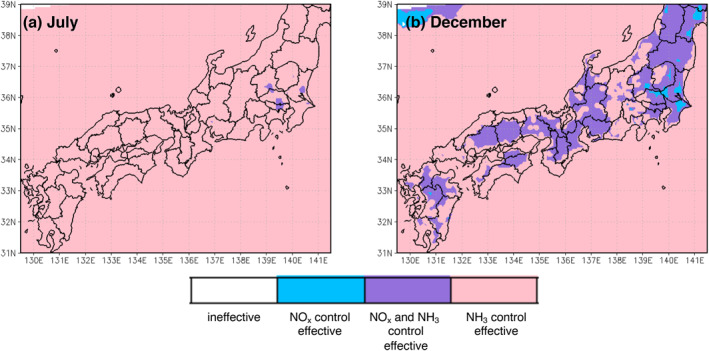
Horizontal distribution of the chemical sensitivity window classification of the particle phase for NO_x_ and NH_3_ emission control in (a) July and (b) December.

## Data Availability

The NHM‐Chem source code is available at subject to a license agreement with the Meteorological Research Institute (2022). The raw data of REASv3.2.1 provided by Kurokawa and Ohara (2020a, 2020b) used for the anthropogenic emission inventory in Northeast Asia can be obtained from https://www.nies.go.jp/REAS/ (last accessed: 23 January 2023). The raw data of GFED v4 used for the biomass burning emission inventory provided by Giglio et al. (2013a, 2013b) can be obtained from https://www.globalfiredata.org/data.html (last accessed: 23 January 2023). The metal emission inventory data of TMI‐Asia v1.1 developed in this study is available from Kayaba (2023a) (https://doi.org/10.17632/r9c59639pg.1). The data of metal content rates in PM2.5 or PM10 used in the TMI‐Asia/Japan v1.1 development is available from Kayaba (2023b) (https://doi.org/10.17632/ygwxphz2p4.1). The observation data of PM2.5 provided by MOE (2023) are available at https://www.env.go.jp/air/osen/pm/monitoring.html (last accessed: 23 January 2023). The map figures in this paper were drawn with GrADS v2.0. The GrADS software provided by Center for Ocean‐Land‐Atmosphere Studies (COLA, 2023) van be obtained at http://cola.gmu.edu/grads/ (last accessed: 14 December 2022).

## Appendix A Effect of Gas–Aerosol Partitioning on Nitrate Change Due To NO_x_ and NH_3_ Emission Control

As described in Section 3.2.3, on‐road NO_x_ and NH_3_ emission changes due to passenger car electrification alone increased H^+^ (decreased pH) in urban areas in July, while the change was random in December (Figure A1). Reducing NO_x_ and NH_3_ will not affect aerosol pH if HNO_3_ and NH_3_ gases are reduced, not the particle phase. Therefore, this seasonal difference in change is discussed in terms of the gas–aerosol partitioning of HNO_3_–NO_3_^−^ and NH_3_–NH_4_^+^.

Figure A2 shows the ratio of concentration change based on passenger car electrification alone. In December, the reduction ratios of the molar concentrations of NH_4_^+^ and NO_3_^−^ were comparable (1–1.1 times), while in July, NH_4_^+^ was reduced more than 2 times than NO_3_^−^ in many areas (Figure A2a). The ratio of the particle phase (NH_4_^+^) reduction to the total ammonium (TNH_4_ = NH_4_^+^ + NH_3_) reduction was comparable in July and December (Figure A2b), whereas for total nitric acid (TNO_3_ = NO_3_^−^ + HNO_3_), the reduction was clearly more from the particle phase (NO_3_^−^) in December than in July (Figure A2c). This means that NO_3_^−^ was less reduced in July than in December, and NH_4_^+^ was reduced more than NO_3_^−^ from the particle phase. The equilibrium constants for NH_3_ + H^+^ → NH_4_^+^ and HNO_3_ → NO_3_^−^ + H^+^ ($H_{{\text{NH}}_{3}}^{*}$ and $H_{{\text{HNO}}_{3}}^{*}$, respectively) decrease faster for $H_{{\text{NH}}_{3}}^{*}$ than for $H_{{\text{HNO}}_{3}}^{*}$ with increasing temperature (Clegg & Brimblecombe, 1990; Clegg et al., 1998; Guo et al., 2018). Then it would be expected that NH_4_^+^ would remain in the particle phase rather than NO_3_^−^ during the summer. Nevertheless, NH_4_^+^ decreased more. In conclusion, this is due to the low pH of the ambient aerosol in summer. The details were described below.

Figure A3 shows the ratio of the particle phase in TNO_3_ or TNH_4_ (εNO_3_ = NO_3_^−^/(NO_3_^−^ + HNO_3_), εNH_4_ = NH_4_^+^/(NH_4_^+^ + NH_3_), respectively) to ambient aerosol pH conditions. The dots are scatter plots of aerosol pH and εNO_3_ and εNH_4_ from the simulation results (2050R experiment). In July, NH_4_ is clearly partitioned into the particle phase and NO_3_ into the gas phase, and the two are almost completely separated (Figure A3a). On the other hands, in December, the gas–aerosol partitioning of NO_3_ and NH_4_ was comparable in most cases (Figure A3b).

Guo et al. (2017) proposed that $\varepsilon {\text{NO}}_{3}^{-}$ and $\varepsilon {\text{NH}}_{4}^{+}$ can be expressed as sigmoid functions with respect to pH by the following Equations A1 and A2, depending on the ambient aerosol pH, LWC, and *T* conditions.

(A1)
$$\varepsilon {\text{NO}}_{3}^{-}=\frac{K_{n1}H_{{\text{HNO}}_{3}}\;C_{ｗ}\;R\;T}{\gamma_{H^{+}}\gamma_{{\text{NO}}_{3}^{-}}\;H_{\text{aq}}^{+}+K_{n1}\;H_{{\text{HNO}}_{3}}C_{ｗ}\;R\;T}$$

(A2)
$$\varepsilon {\text{NH}}_{4}^{+}=\frac{\frac{\gamma_{H^{+}}}{\gamma_{{\text{NH}}_{4}^{+}}}\frac{H_{{\text{NH}}_{3}}}{K_{a}}H_{\text{aq}}^{+}\;C_{ｗ}\;R\;T}{1+\frac{\gamma_{H^{+}}}{\gamma_{{\text{NH}}_{4}^{+}}}\frac{H_{{\text{NH}}_{3}}}{K_{a}}H_{\text{aq}}^{+}\;C_{ｗ}\;R\;T}$$

where $H_{\text{aq}}^{+}$ is the concentration of hydronium ions in the aerosol aqueous phase (mol L^−1^), *C*_*w*_ is the LWC in air (μg m^−3^), *K*_*n*1_ and *K*_*a*_ are the acid dissociation constants for HNO_3_ and NH_4_^+^, respectively (*K*_*n*1_ = 12, *K*_*a*_ = 5.69 × 10^−10^), *R* is the gas constant, and *T* is the temperature (*K*). $H_{{\text{HNO}}_{3}}$ and $H_{{\text{NH}}_{3}}$ are Henry’s Law constants for HNO_3_ and NH_3_, which are functions of temperature. $\gamma_{H^{+}}$, $\gamma_{{\text{NO}}_{3}^{-}}$, $\gamma_{{\text{NH}}_{4}^{+}}$ are the ion activity coefficients of H^+^, NO_3_^–^, and NH_4_^+^ respectively. $\gamma_{H^{+}}$ $\gamma_{{\text{NO}}_{3}^{-}}=0.324$ and $\gamma_{{\text{NH}}_{4}^{+}}$ $\gamma_{{\text{NO}}_{3}^{-}}$ = 0.017, in this study.

The S‐curves in Figure A3 are $\varepsilon {\text{NO}}_{3}^{-}$ and $\varepsilon {\text{NH}}_{4}^{+}$ derived from theoretical Equations A1 and A2. $\varepsilon {\text{NO}}_{3}^{-}$ increases with higher ambient aerosol pH due to promoted particulation. The S‐curve of $\varepsilon {\text{NO}}_{3}^{-}$ shifts to the right as gasification is promoted at higher temperatures and lower humidity, even under the same pH conditions. $\varepsilon {\text{NH}}_{4}^{+}$, contrary to $\varepsilon {\text{NO}}_{3}^{-}$, tends to be higher at lower pH and tends to shift to the left at higher temperatures and lower humidity. The gas–aerosol partitioning of the simulation results is consistent with the function derived in the theoretical equation; that is, it can be approximately explained by aerosol pH, LWC, and temperature.

Nenes et al. (2020) proposed a new framework to evaluate whether NO_x_ and NH_3_ emission controls are effective in reducing particle phase concentrations by a chemical sensitivity window delimited by $\varepsilon {\text{NO}}_{3}^{-}$ and $\varepsilon {\text{NH}}_{4}^{+}$ thresholds. In Figure A3, it can be considered that controlling primary emissions is not effective in reducing aerosol mass when the particle fraction is low. When the threshold value of $\varepsilon {\text{NO}}_{3}^{-}$ (or $\varepsilon {\text{NH}}_{4}^{+}$) = 0.3 was used in this study, the pH was uniquely determined from the sigmoid function, and when it is applied to various LWC widths, Figure A4 was obtained. In this way, the four chemical sensitivity windows derived can be classified as 1: NO_x_ control effective, 2: NH_3_ control effective, 3: both NO_x_ and NH_3_ control effective, and 4: NO_x_ nor NH_3_ control ineffective.

In July, most of the target data (model simulation result data for the grid with $\Delta {\text{pH}}_{H^{+}}$ > 0.05 in Figure A1) are classified into chemical window 2 (Figure A4a), consistent with the positive trend of $\Delta {\text{pH}}_{H^{+}}$ in Figure A1a. The blue line is located higher in July than in December due to higher temperatures, and NO_x_ emission control is ineffective, except for the case of higher aerosol pH. And indeed, because of the low aerosol pH in summer (the plot is located lower than in December), many were not classified in chemical window 1 or 3 (Figure A4a). In terms of the high humidity and high LWC characteristics in summer, emission controls are more effective in reducing particle phase concentrations (the plots are more right side than in December), and part of the plots was classified as chemical window 3. In the horizontal distribution of chemical windows, the area of no $\Delta {\text{pH}}_{H^{+}}$ sensitivity in Tokyo and Saitama corresponded to chemical window 3 (Figures A1a and A5a), suggesting that the effects of acid and base reduction due to decreases in both NO_3_^−^ and NH_4_^+^ were offset.

In December, many of the target data were evaluated to be classified in chemical window 3, and some were classified in windows 1 and 2. This is also consistent with the random trend of $\Delta {\text{pH}}_{H^{+}}$ in Figure A1b. With similar rates of NO_3_ and NH_4_ as particle phases (Figure A3b), the offsetting effects of NO_3_^−^ and NH_4_^+^ reduction occur in many grids. In December, the positive and negative distribution of $\Delta {\text{pH}}_{H^{+}}$ did not correspond to the horizontal distribution of chemical window classification (Figures A1b and A5b). This framework is only a sensitivity classification for aerosol mass, so the classification might be slightly different if the ionization of NO_3_^−^ and NH_4_^+^ is considered.

These results suggested that NO_3_ tends to exist in the gas phase and NH_4_ in the particulate phase during the summer due to the low pH and that the effect of NH_3_ reduction was dominant despite the reduction of both NO_x_ and NH_3_ as a result of the chemical sensitivity window classification.
