# Supplementary material for: Potential Impacts of Energy and Vehicle Transformation Through 2050 on Oxidative Stress‐Inducing PM2.5 Metals Concentration in Japan
Source: Geohealth. 2023 Oct 13;7(10):e2023GH000789. doi: 10.1029/2023GH000789 (PMC10574721; doi:10.1029/2023GH000789)
Supplement: Supplementary file 1 — Supporting Information S1 [file GH2-7-e2023GH000789-s001.pdf]

GeoHealth

Supporting Information for

Potential Impacts of Energy and Vehicle Transformation through 2050 on Oxidative Stress-Inducing $PM_{2.5}$ Metals Concentration in Japan

Satoko Kayaba[1,2] and Mizuo Kajino[2,3]

1 Graduate School of Science and Technology, University of Tsukuba, Tsukuba, Ibaraki 305-8572, Japan

2 Meteorological Research Institute, Japan Meteorological Agency, Tsukuba, Ibaraki 305-0052, Japan

3 Faculty of Life and Environmental Sciences, University of Tsukuba, Tsukuba, Ibaraki 305-8572, Japan

Contents of this file

- Text S1 to S6
- Figures S1 to S19
- Tables S1 to S7

Introduction

This supporting information provides the following contents.

- The details of model assumptions (Table S1)
- The details of the revision of the transition metal emission inventory (Text S1, Figure S1–S4, Table S2–S3)
- Uncertainty in aerosol pH due to disregarded organic matter (Text S2, Figure S5)
- Assumptions for the lightweighting of the vehicle body frame and the drive battery through 2050 (Text S3, Table S4–S5)
- The prediction of the share of passenger car sales by vehicle type through 2050 (Figure S6)
- The vehicle scrap and survival ratio according to vehicle age (Figure S7)
- The improvement of the energy consumption of vehicles through 2050 (Table S6)
- Energy mix and diurnal electricity supply-demand pattern in 2050 (Text S4, Figure S8)
- Surplus electricity of solar energy and electricity demand for BEVs and PHEVs charging in 2050 (Text S5)
- Comparison of observation and modeling for $PM_{2.5}$ ionic components, temperature and RH (Figure S9–S10, Table S7)
- Seasonal variation of the concentration, emission, and deposition of $PM_{2.5}$-Fe, Cu, and Zn (Figure S11, Figure S12) and the contribution of continental Northeast Asian emissions to the monthly average concentration of $PM_{2.5}$ Fe, Cu, and Zn (Figure S13–15)
- The sensitivity of $PM_{2.5}$-NO_3^- concentration in July by renewable energy shifting (Figure S16)
- The aerosol pH sensitivity in the 2050R&E&L experiment in December (Figure S17)
- The relationship between aerosol pH and the water-soluble fraction of metals (Figure S18)
- The concentrations of water-soluble metals in the BASE experiment and the sensitivity in the 2050R&E&L experiment (Figure S19)

Table S1. Simulation setting in this study.

Model	Offline NHM-Chem[a] (Kajino et al., 2019a; 2020; 2021)		
Region	Domain 1: East Asia (dx = 30 km)<br>Domain 2: Japan from Kyusyu to Tohoku (dx = 6 km)		
Period	From January 1 to December 31, 2015		
Boundary conditions of NHM	Reanalysis data<br>Domain 1: JRA-55, 6 hourly (Kobayashi et al., 2015)<br>Domain 2: JMA's Meso-Regional Objective Analysis (MA), 3 hourly (https://www.jma.go.jp/jma/jma-eng/jma-center/nwp/nwp-top.htm, last accessed: 20 January 2023).		
Boundary conditions of CTM[b]	Domain 1: monthly climatological data simulated by a global model<br>Gases : MRI-CCM2 (Deushi and Shibata, 2011)<br>Aerosols: MASINGAR-mk2 (Tanaka et al., 2003; Yumimoto et al., 2017)<br>Domain 2: Domain 1		
Time intervals	1 h (output of NHM and input/output of CTM)		
Chemical species	gaseous and particle pollutants excluding metals		metals
CTM option	Option1015[c]		Option900[d]
Emission	Anthropogenic	East Asia: REASv3.2.1 (2015 base) (Kurokawa and Ohara, 2020)<br>*updated from v3.2 in Dec 2021.<br><br>Japan: PM2.5EI (2012 base) (Morikawa, 2017)<br>+ EAGrid for navigation (2010 base) (Fukui et al., 2014; Kannari et al., 2007)	TMI-Asia , TMI-Japan v1.1<br><br>*v1.1 was modified from v1.0 (Kajino et al., 2020) in this study. See Text S1.
	Biomass burning	GFED v4 (Giglio et al., 2013)	
	Biogenic VOC	MEGAN v2 (Guenther et al., 2006)	
	Volcano SO_2	Observation data of JMA	
	Mineral dust	Inline calculation based on the method of Han et al. (2004)	
	Sea salt	Inline calculation based on the method of Clark et al. (2006)	

a. NHM-Chem is coupled with the meteorological model NHM offline or online. In the case of offline coupling, drive NHM first and then the chemical transport model using the results of the meteorological simulation.
b. Chemical transport model (CTM) part of NHM-Chem
c. Consider gas-phase, liquid-phase, photolysis, and heterogeneous reactions of major gases in the troposphere, microphysical processes in aerosols, wet and dry deposition. Aerosol representation is 5-category nonequilibrium (Aitken, soot-free accumulation, soot-containing accumulation, dust, and sea salt) (Kajino et al., 2019a; 2021).
d. Chemical reactions, aerosol aggregation, and condensation were not considered. This option was developed to simulate the transport of chemically inert, such as radionuclides (Kajino et al., 2019b) and transition metals (Kajino et al., 2020)

Text S1. Revision of transition metal emission inventory; TMI-Asia/Japan v1.1

In this study, metal concentrations were simulated using the transition metal emission inventory TMI-Asia/TMI-Japan. Figure S1a, 1c, and 1e show the scatter plots of the BASE experiment results calculated using TMI-Asia/Japan v1.0 and the observations. For Fe, the model is consistent with observations, but the model overestimates Cu significantly, and it overestimates Zn at the background sites. The overvaluation of Cu was also pointed out as a future issue in the v1.0 development paper (Kajino et al., 2020).

Therefore, in this study, emission factors (EF) for Fe, Cu, and Zn were revised for the major sources, vehicle brakes and the industrial sector, with a modification from v1.0. As a result, the reproducibility of Fe was not impaired, while the reproducibility of Cu and Zn was improved (Figure S1b, 1d, and 1f, Table S2). The modification methods are (1) EF modification for brake wear-derived metals, (2) EF modification for industrial sector-derived metals, (3) new consideration of metals from road wear and resuspension PM in Japan, and (4) new consideration of metals from all non-exhaust PM (tire, brake, and road wear, and resuspension) in Asia region.

In this section, an overview of TMI-Asia/TMI-Japan v1.0 is first provided (Text S1.1). The basis and details of each method are then explained (Text S1.2 - S1.5). Finally, we show the change in reproducibility before and after the improvement (Text S1.6). Although only the $PM_{2.5}$ particle size is discussed in the main text, the PM_{10} particle size is also described in the supporting information.

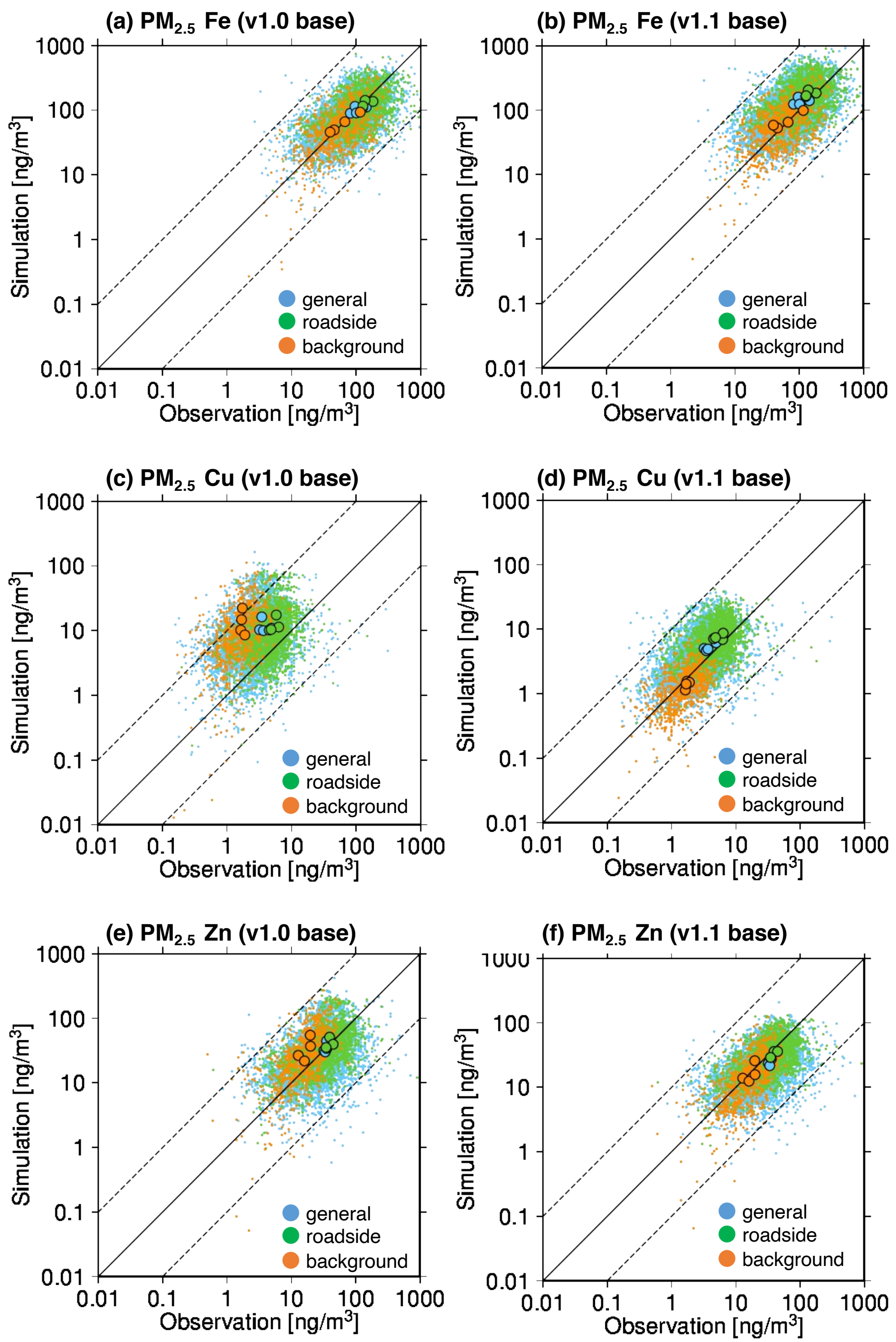

Figure S1. Scatter plots of model's BASE experiment results and observation data for $PM_{2.5}$-Fe, Cu, and Zn in 2015. (a), (c), and (e) are the result of based on TMI-Asia/Japan v1.0, (b), (d), and (f) are based on v1.1.The small dots are daily data (192 site nationwide × 56 days, excluding the missing days). The large dots are seasonal data (4 seasons × 3 site categories).
The solid line indicates 1:1, and the dashed lines indicate a factor of 10.

Table S2. Statistical comparison of model's BASE experiment result and observation data for $PM_{2.5}$-Fe , Cu, and Zn in 2015. The difference between the results of the original (v1.0) and modified (v1.1) inventories are shown.

Species	Inventory modification	Site category	N	Obs. mean (ng/m^3)	Sim. mean (ng/m^3)	R	MB (ng/m^3)	NMB (%)
$PM_{2.5}$ Fe	Before (Based on TMI-Asia/Japan v1.0)	all site	7267	112.2	104.6	0.39	-7.6	-7
		general	5052	107.1	102.4	0.40	-4.7	-4
		roadside	1639	144.6	126.3	0.31	-18.3	-13
		background	576	64.5	62.4	0.51	-2.1	-3
	After (Based on TMI-Asia/Japan v1.1)	all site	7267	112.2	142.2	0.36	30.0	27
		general	5052	107.1	138.3	0.35	31.2	29
		roadside	1639	144.6	180.6	0.27	36.0	25
		background	576	64.5	67.4	0.50	2.9	4
$PM_{2.5}$ Cu	Before (Based on TMI-Asia/Japan v1.0)	all site	7267	4.1	12.2	0.07	8.2	200
		general	5052	3.9	12.0	0.06	8.1	210
		roadside	1639	5.5	12.5	0.11	7.1	130
		background	576	1.7	13.6	0.33	11.8	680
	After (Based on TMI-Asia/Japan v1.1)	all site	7267	4.1	5.4	0.20	1.4	33
		general	5052	3.9	5.2	0.17	1.3	34
		roadside	1639	5.5	7.5	0.14	2.0	36
		background	576	1.7	1.4	0.35	-0.3	19
$PM_{2.5}$ Zn	Before (Based on TMI-Asia/Japan v1.0)	all site	7267	33.9	36.4	0.20	2.5	7
		general	5052	34.3	35.4	0.18	1.1	3
		roadside	1639	38.6	40.5	0.25	1.9	5
		background	576	17.1	34.0	0.51	16.9	99
	After (Based on TMI-Asia/Japan v1.1)	all site	7267	33.9	26.3	0.25	-7.6	-22
		general	5052	34.3	25.4	0.20	-8.9	-26
		roadside	1639	38.6	32.8	0.30	-5.8	-15
		background	576	17.1	16.4	0.50	-0.6	-4

Text S1.1. Outline of TMI-Asia/Japan v1.0

TMI-Asia/Japan v1.0 is an emission inventory of transition metals developed by Kajino et al. (2020). These are for Northeast Asia (0.25° × 0.25°) and Japan (1 km × 1 km), respectively, for 10 metals (Fe, Cu, Mn, Co, V, Ni, Pb, Zn, Cd, and Cr) in 3 categories (anthropogenic $PM_{2.5}$ particle size, anthropogenic PM_{10} particle size, and Asian dust PM_{10}). Anthropogenic metal emissions are derived in Equation (1).

$$E_{metal} = \sum_{i=1}^{nv} \left(E_{PM,i} \times C_{metal,i}\right) \qquad (1)$$

where E_{metal} is the emission flux of metal (g-metal m^{-2} sec^{-1}). $E_{PM,i}$ is the emission flux (g-PM m^{-2} sec^{-1}) of $PM_{2.5}$ or PM_{10} for each sector of total nv species, for Northeast Asia based on REAS v2 (Kurokawa et al., 2013, 0.25°× 0.25°, base year = 2008) and for Japan based on PM2.5EI and EAGrid combined data. $C_{metal,i}$ is the metal content (g-metal g-PM^{-1}) in the PM emission flux for each sector. They are based on SPECIATE v4.4 database values provided by US EPA. In SPECIATE, multiple subsectors (e.g., "coal power plants", "natural gas power plants", etc. for the "power plat" sector) and multiple literature values are registered for each sector. The average of these values was used. For more details, please refer to the development paper (Kajino et al., 2020).

Text S1.2. Modification of metal content in PM from brake wear in v1.1

Table S3 shows the metal content of brake wear used for the development of TMI-Asia/Japan v1.0 and the modified values in this study.

In v1.0, the metal content of the brake wear was very different between $PM_{2.5}$ and PM_{10} particle sizes for Fe, Cu, and Zn, with very low assumptions for Cu and Zn in the $PM_{2.5}$ particle size (0.04% and 0.03% for Cu and Zn, respectively) (Table S3). This is because there was only one literature value in SPECIATE v4.4 that measured the metal content of the $PM_{2.5}$ particle size brake wear (Hildemann et al., 1991), which was based on iron-main semi-metallic material, and therefore, the values for Cu, and Zn contents were very low. On the other hand, various brake pad material-based literature values were registered for PM_{10}, which led to differences in metal content between $PM_{2.5}$ and PM_{10} particle sizes.

Semi-metallic is often used for HDVs such as trucks, while non-asbestos organics (NAO) is the most common brake pad material for passenger cars in Japan. Therefore, in v1.1, the metal content was derived by weighted averaging the brake wear PM emission ratios for passenger cars and heavy-duty vehicles to the literature values of metal content in brake dust for NAO and semi-metallic materials (Hagino et al., 2016; Garg et al., 2000). The brake wear PM emissions for passenger cars and heavy-duty vehicles were calculated based on traffic volumes and ECLIPSE emission factors.

Furthermore, a part of the brake wear generated is deposited on the friction surfaces and the car body, and the ratio of suspended in the air is approximately 20%–90% (Garg et al., 2000; Sanders et al., 2003; Iijima et al., 2008; Hagino et al., 2016). Although discrepancies exist among literature values, it was assumed that approximately 55%, which is the average of them, were airborne in this study. As a result, the contents of $PM_{2.5}$-Fe, PM_{10}-Fe, $PM_{2.5}$-Cu, PM_{10}-Cu, $PM_{2.5}$-Zn, and PM_{10}-Zn in brake wear were revised to 22.2%, 22.1%, 1.5%, 1.6%, 1.3%, and 1.4%, respectively.

Table S3. Metal content (%) of PM from Industry, vehicle-brake, tire and road wear and resuspension used to develop the TMI inventory. Differences between the assumptions used in the development of the original (v1.0) and modified inventory developed in this study (v1.1) are shown.

Sector	Region	Species	Metal content rate (g-metal/g-PM, in weight %)		Modification method
			before (v1.0)	**after (v1.1)**	
Industry	TMI-Asia	$PM_{2.5}$ Cu	1.01	**0.024**	Before modification (v1.0):<br>the "average value" of the metal content rate in PM from various industrial subsectors registered in SPECIATE v4.4 was used.<br><br>After modification (v1.1):<br>the "median value" of these was used.
		PM_{10} Cu	1.24	**0.023**	
		$PM_{2.5}$ Zn	2.38	**1.00**	
		PM_{10} Zn	2.99	**1.05**	
Industry non-ferrous metals	TMI-Japan	$PM_{2.5}$ Cu	2.37	**1.01**	
		PM_{10} Cu	2.74	**0.28**	
		$PM_{2.5}$ Zn	6.66	**1.38**	
		PM_{10} Zn	7.54	**1.42**	
Vehicle-brake wear	TMI-Japan[a]	$PM_{2.5}$ Fe	11.5	**22.2**	Before modification (v1.0):<br>the average of multiple literature values registered in SPECIATE v4.4 was used for $PM_{2.5}$ and PM_{10} particle sizes, respectively.<br><br>After modification (v1.1):<br>Literature values of metal content in brake dust for NAO[b] (Hagino et al., 2016) and semi-metallic[c] materials (Garg et al., 2000) were used. They were weighted and averaged by the ratio of brake wear PM emissions of passenger cars and heavy-duty vehicles in Japan.<br>In addition, the proportion suspended in the atmosphere was taken into account.
		PM_{10} Fe	41.0	**22.1**	
		$PM_{2.5}$ Cu	0.04	**1.50**	
		PM_{10} Cu	4.28	**1.62**	
		$PM_{2.5}$ Zn	0.03	**1.34**	
		PM_{10} Zn	1.41	**1.41**	
Vehicle-road wear and resuspension	TMI-Japan[a]	$PM_{2.5}$ Fe	-	**3.0**	Before modification (v1.0):<br>Unconsidered. Because EAGrid does not estimate $PM_{2.5}$ and PM_{10} emissions from road wear and resuspension.<br><br>After modification (v1.1):<br>Newly considered. The $PM_{2.5}$ and PM_{10} emissions from road wear and resuspension were assumed to be 0.65/0.35 of those of brake wear based on EMEP/EEA (2019) estimates.
		PM_{10} Fe	-	**3.0**	
		$PM_{2.5}$ Cu	-	**0.03**	
		PM_{10} Cu	-	**0.03**	
		$PM_{2.5}$ Zn	-	**0.1**	
		PM_{10} Zn	-	**0.1**	
Vehicle-tire wear	TMI-Japan[a]	$PM_{2.5}$ Zn	0.04	**1.02**	Before modification (v1.0):<br>the average of multiple literature values registered in SPECIATE v4.4 was used for $PM_{2.5}$ and PM_{10} particle sizes, respectively.<br><br>After modification (v1.1):<br>The same value as PM_{10} was applied to $PM_{2.5}$.
		PM_{10} Zn	1.02	**1.02**	

a. The metal emissions from brake and tire wear newly added to TMI-Asia using these values of TMI-Japan (previously not considered).

b. Non-asbestos organics; mainly composed of organic compounds, mineral fibers, and graphite. This material is mainstream for passenger cars in Japan.

c. Approximately 30%–60 % of mass is composed of metals (mainly steel and iron). This material is often applied to heavy vehicles because of its high durability.

Text S1.3. Modification of metal content in PM from Industry sector

The overestimation of Cu and Zn at the background sites (Figure S1c and S1e, Table S2) suggests that continental transport is excessive. In TMI-Asia v1.0, the industrial sector was the dominant source of Cu and Zn emissions in the Northeast Asian region (accounting for 98% of them). Based on SPECIATE v4.4, the Cu and Zn content in $PM_{2.5}$ is very small (＜1%) in more than 90% of industrial species. However, if the metal content is derived as an "average" of several subsectors, some subsectors with high Cu and Zn emission rates (related to Cu and Zn smelting) will raise the average value. This is considered unrepresentative. Therefore, in v1.1, the median value was used instead of the mean value. As a result, the content of $PM_{2.5}$-Cu, PM_{10}-Cu, $PM_{2.5}$-Zn, and PM_{10}-Zn from the industrial sector in TMI-Asia were modified to approximately 1/40, 1/50, 1/2, and 1/3, respectively (compared to the v1.0 case in Table S3). The same treatment was applied to TMI-Japan.

Text S1.4. Development of road and resuspension emission inventory in Japan

Since EAGrid does not estimate PM emissions from road wear and resuspension, TMI-Japan v1.0 did not consider metal emissions derived from them. In TMI-Japan v1.1, road wear and resuspension-derived metal emissions are newly considered for Fe, Cu, and Zn only. This ratio is based on the ratio of the EMEP/EEA (2019) estimates.

First, $PM_{2.5}$ and PM_{10} emissions from road wear and resuspension were assumed to be 0.65/0.35 of those from brake wear. Then, they were multiplied by the metal content described in Table S3. The mass fractions of Fe, Cu, and Zn in road wear and resuspension PM were assumed to be 3.0, 0.03, and 0.1%, respectively, based on literature values registered in SPECIATE.

Text S1.5. Development of non-exhaust PM emission inventory in Northeast Asia (tire, brake and road wear, and resuspension)

Because REAS does not estimate the emissions of non-exhaust PM from road traffic, TMI-Asia v1.0 did not consider metal emissions from all non-exhaust PM (tire, brake, and road wear, and resuspension), but v1.1 newly considers them.

First, non-exhaust PM (tire, brake and road wear and resuspension) emission fluxes from vehicles were estimated (Figure S2a and S2c). Then, they were multiplied by the metal content described in Table S3. The non-exhaust PM emission was derived by multiplying the vehicle activity assumption (km km^{-2} sec^{-1}) from REAS v1.0 by the EF (mg-PM km^{-1}) for non-exhaust PM from ECLIPSE, a global-scale emissions inventory (see Table S4.5 in Klimont et al., 2017).

Since the only available data on vehicle activity was REAS v1.0 (base year = 2005), we adjusted the data for China only by a factor of 5 (the growth rate of the number of registered vehicles in China between 2005 and 2015). No correction was applied to Japan and South Korea as the number of vehicles owners has not substantially changed. It is desirable to update in the future with REAS v3-based vehicle activity data. The estimated non-exhaust PM emissions (Figure S2a and S2c) are approximately an order of magnitude smaller than those of exhaust PM (Figure S2b and S2d) in the China region, suggesting that the contribution of non-exhaust PM to metal emissions is not as large as that in Japan. The difference between the totalized values of brake dust emissions in the Japanese region derived by this method and that of EAGrid values was approximately 7%. Therefore, the values derived by this method were reasonable.

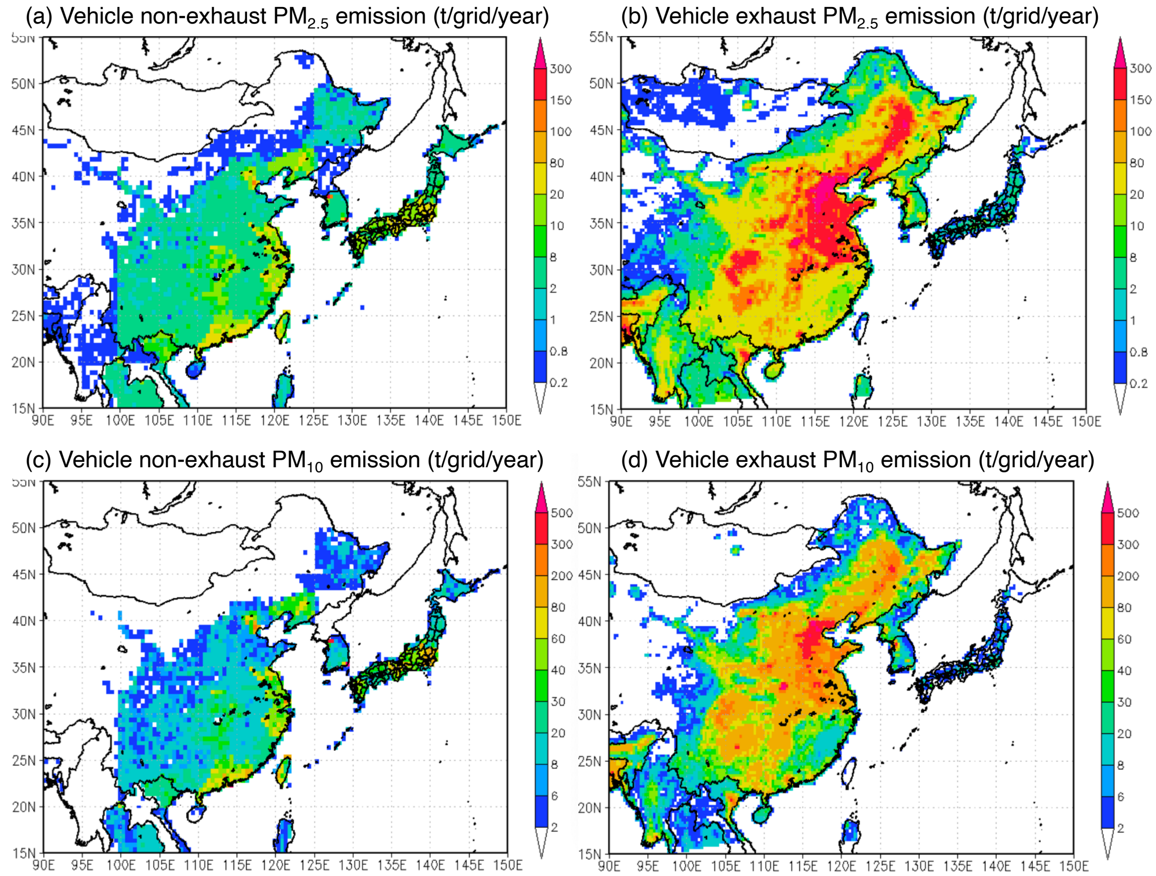

Figure S2. $PM_{2.5}$ and PM_{10} emissions from road transport in Northeast Asia. (a) and (c) show non-exhaust PM emission (tire, brake, and road wear, and resuspension) estimated in this study. (b) and (d) show exhaust PM emissions based on REAS v3.2.

Text S1.6. Model evaluation result (difference in reproducibility based on TMI-Asia/Japan v1.0 or v1.1)

Figure S3 and Figure S4 show the annual primary emissions of anthropogenic Fe, Cu and Zn in Northeast Asia and Japan. The differences between the original inventory (v1.0) and the revised one (v1.1) are shown.

In the Asian region, Cu and Zn emissions were revised downward significantly due to the revised emission factors for the industrial sector (Figure S3c and S3e). The newly added brake dust-derived metals did not account for such a large proportion of the total compared to other sectors, unlike the case in Japan (3%, 4%, 7%, 11%, 0.5%, and 1% for $PM_{2.5}$-Fe, PM_{10}-Fe, $PM_{2.5}$-Cu, PM_{10}-Cu, $PM_{2.5}$-Zn and PM_{10}-Zn, respectively, as shown in Figure S3b, S3d and S3f).

In Japan, the $PM_{2.5}$ particle size emissions of Fe, Cu, and Zn were raised and those of PM_{10} particle size were lowered (Figure S4a, S4c, and S4e) by modifying the assumption of the metal content of brake dust metals. Emissions from the industrial sector also decreased by 10%–20%, but the impact was not significant in Japan, where the brake-derived contribution was larger, unlike the case in Asia.

The reproducibility of the simulations before and after the modification is shown in Figure S1 and Table S2. $PM_{2.5}$-Cu was overestimated by NMB approximately 100%–200% compared to the observed values in the “general” and “roadside” sites before the correction, but they were reduced to approximately 30%, suggesting that emissions from brake wear were improved. In addition, approximately 700% of the NMBs at the "background" site were reduced to –30%, suggesting that emissions from the industrial sectors in the Northeast Asia were improved. The same verification for PM_{10} was not conducted because there is no nationwide observed data of metal concentrations like that for $PM_{2.5}$. Therefore, the reproducibility of PM_{10} will be verified in a future study.

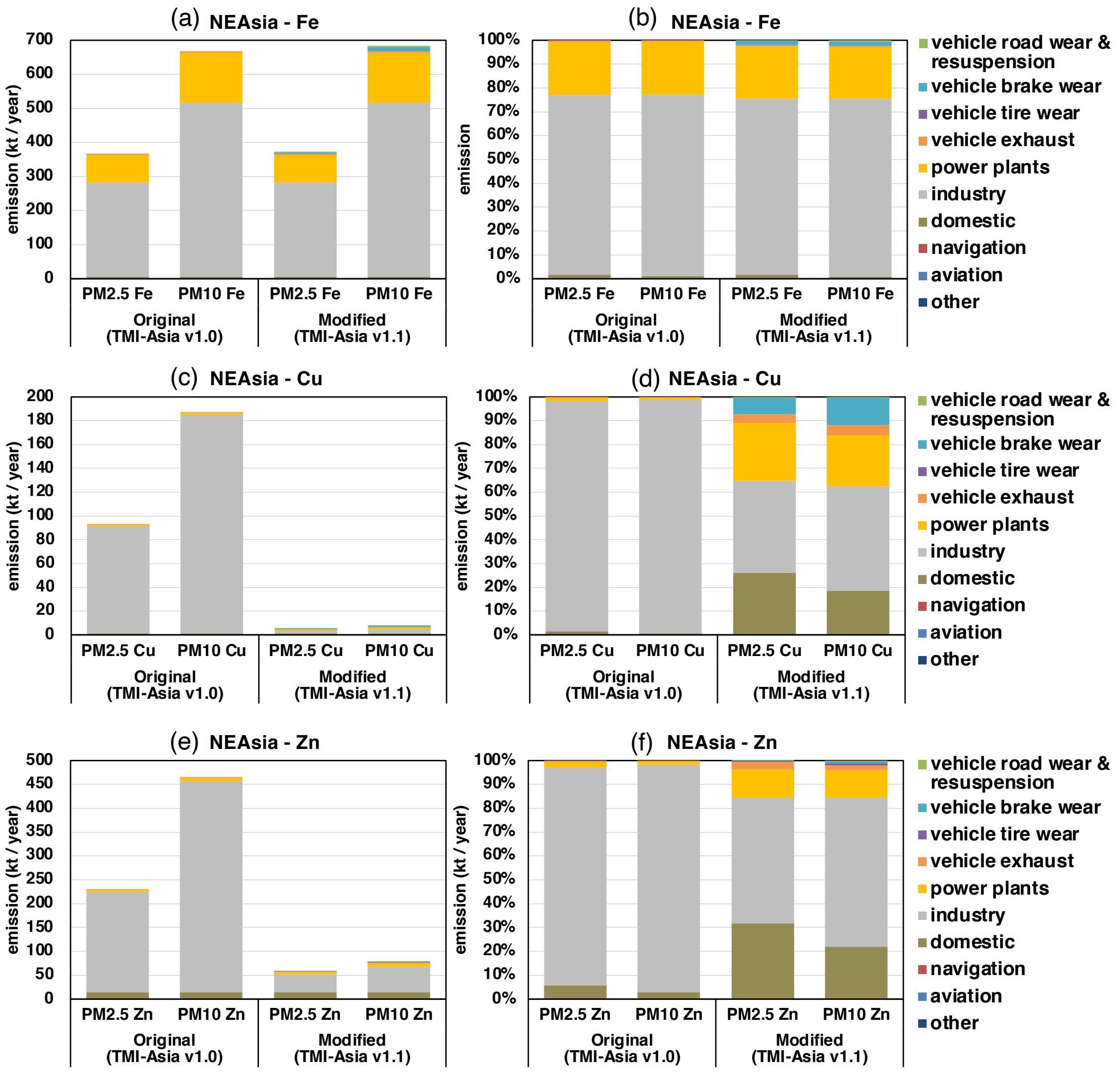

Figure S3. Annual anthropogenic Fe, Cu, and Zn emissions in entire of Northeast Asia (right column) and their sector ratio (right column).The differences between the original inventory (TMI-Asia v1.0) and the modified inventory in this study (v1.1) are shown.

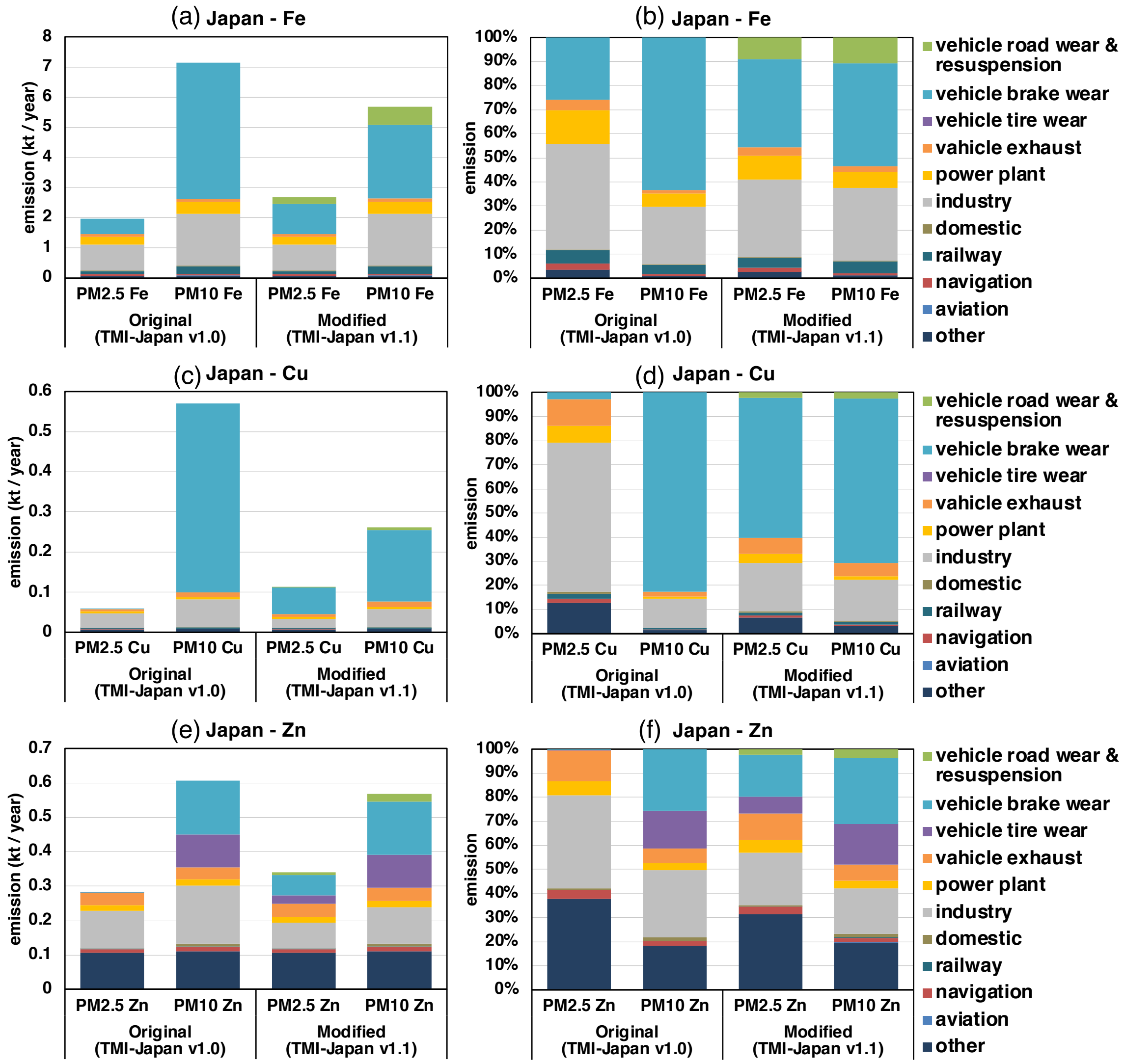

Figure S4. Same as Figure S3 but for TMI-Japan.

Text S2. Uncertainty in aerosol pH due to disregarded organic matter

The contribution of organic matter to aerosol pH was not considered in this study. The hygroscopicity of organic matter raises pH by increasing the LWC. The organic acids decrease pH by increasing H^+. However, the degree of their effect is considered to be small and were ignored in many studies, as discussed in Section 2.2.

Several studies evaluating the effects of organic acids on pH will be introduced. The effect of the LWC derived from organic matter (denoted as LWC_o) on pH is simply defined as $-\log_{10}(1-\varepsilon LWC_o)$, as shown in Figure S5 (where εLWC_o is the fraction of organic matter derived from the total LWC). If εLWC_o is 90%, the pH increases by 1 (Guo et al., 2015). The range of $\varepsilon LWC_o = 0.29 \sim 0.39$ throughout all seasons at several sampling sites in Georgia, USA, and the impact on aerosol pH was estimated to range from +0.15 to +0.23 (Guo et al., 2015). During the 2014 winter haze period in northern China, $\varepsilon LWC_o = 0.12$ and the contribution to pH was estimated to be +0.05 (Song et al., 2018). Another study estimated $\varepsilon LWC_o = 0.48$ in the 2017 Chinese winter haze (Xu et al., 2020), which corresponds to a pH+ of approximately 0.3. Song et al., (2018) also considered the oxalate concentration as 20% of the sulfate concentration as a substitute for total organic acids, resulting in a pH decrease of approximately 0.07 compared to no consideration. Based on these studies, the pH uncertainty due to the unaccounted for organic matter is considered to be on the order of pH 0.01–0.1. Song and Osada (2020) mentioned that the oxalic acid concentration in Nagoya, Japan, (1.08 nmol m^{-3}) was low compared to other cities, such as Chengdu, China, (6.1–5.3 nmol m^{-3}), and the effect of organic acids on pH would be small.

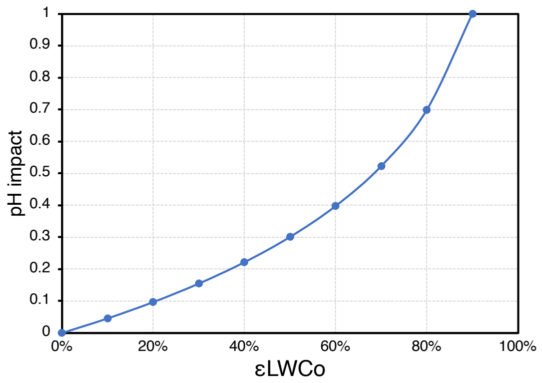

Figure S5. Impact on pH as εLWC_o changes.

Text S3. Assumptions for the lightweighting of the vehicle body frame and drive batteries through 2050.

Table S4 shows the lightweighting estimation of the body frame for each vehicle segment (based on Islam et al., 2020, Table 6). The average weights of the compact, midsize, and small SUV vehicle segments were used, since this study focuses on passenger cars in the Japanese market. The light-weighting of the body frame will progress relatively early (approximately –10% achieved by 2025). Thereafter, it will gradually decrease, and by 2050 it will be approximately 20% lighter than that in 2015.

Table S5 shows the estimated energy density improvement of lithium-ion batteries (based on Islam et al., 2020, Table 5). The current battery performance is based on the data provided by ANL from suppliers (Islam et al., 2020). Assuming the same driving range, the inverse of the energy density is expressed as the reduction ratio of the battery mass. The battery weight per kWh will be approximately 43% lighter in 2050 than in 2015.

Table S4. Lightweighting of the body frame through 2050 (Islam et al., 2020, Table 6).

Segment	Technology progress[a]	Productionn year[b]				
		2020	2025	2030	2035	2050
Compact	Low	977	938	928	928	928
	High	977	870	801	791	791
Midsize	Low	1194	1098	1075	1075	1075
	High	1194	1003	896	836	812
Small SUV	Low	1205	1121	1085	1036	988
	High	1205	1060	988	940	868
Midsize SUV	Low	1246	1109	1084	1034	984
	High	1246	1084	997	947	872
Pickup truck	Low	1388	1221	1194	1152	953
	High	1388	1221	1097	1055	999
Average of compact, midsize and small SUV (kg)	Low	1125	1052	1029	1013	997
	Average	1125	1015	962	934	910
	High	1125	978	895	856	824
**body frame lightweighting ratio from 2020 (%)**	**Low**	**-0.0**	**-6.5**	**-8.6**	**-10.0**	**-11.4**
	**Average**	**-0.0**	**-9.8**	**-14.5**	**-17.0**	**-19.1**
	**High**	**-0.0**	**-13.1**	**-20.5**	**-24.0**	**-26.8**

Table S5. Energy density improvement of Li-ion battery through 2050 (Islam et al., 2020, Table 5).

Segment	Technology progress[a]	Productionn year[b]				
		2020	2025	2030	2035	2050
Energy density (Wh/kg)	Low	170	170	230	240	280
	Average	170	200	270	280	300
	High	170	230	310	320	320
Battery weight (kg/kWh)	Low	5.88	5.88	4.35	4.17	3.57
	Average	5.88	5.12	3.79	3.65	3.35
	High	5.88	4.35	3.23	3.13	3.13
**Battery lightweighting ratio from 2020 (%)**	**Low**	**-0.0**	**-0.0**	**-26.1**	**-29.2**	**-39.3**
	**Average**	**-0.0**	**-13.0**	**-35.6**	**-38.0**	**-43.1**
	**High**	**-0.0**	**-26.1**	**-45.2**	**-46.9**	**-46.9**

a. " Low" is the business-as-usual scenario, " High" is the aggressive technology advancement scenario
b. These various technologies were assessed for six different timeframes: laboratory years 2015 (reference), 2020, 2025, 2030, and 2045. A delay of 5 years was assumed between the laboratory year and the production year, so the starting year for the estimates was 2020.

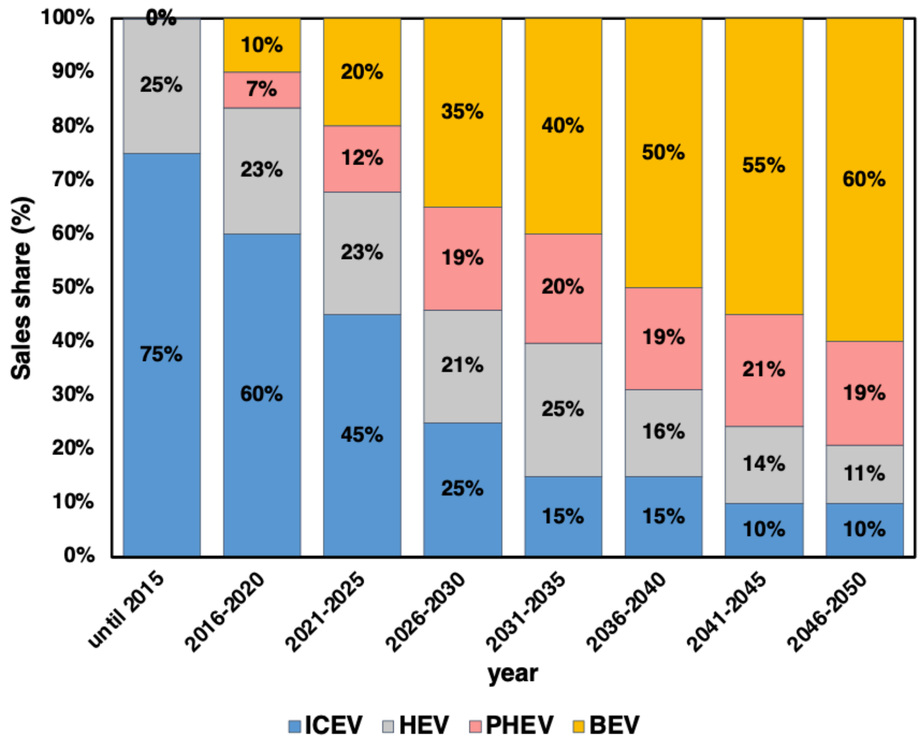

Figure S6. Prediction of the share of passenger car sales by vehicle type through 2050 in the Japanese market. These values were obtained from Fig. 4 of Sato and Nakata (2019), estimated based on the target and the data of the Ministry of the Environment, the Japan Automobile Manufacturers Association, and the Next-Generation Vehicle Promotion Center. This study assumes that the ratio changes every five years and does not consider FCVs.

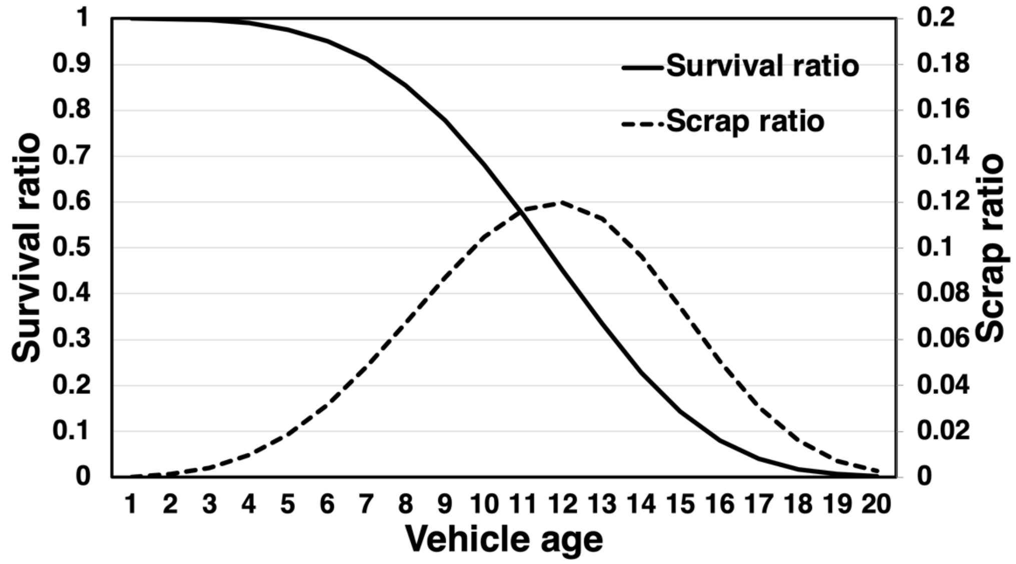

Figure S7. Vehicle scrap and survival ratio according to vehicle age.

Table S6. Energy consumption improvement through 2050 (Islam et al., 2020, Figure 11, 13, and 15). The energy consumption of PHEVs was assumed to be a composite of the gasoline consumption of HEVs (30%) and the electricity consumption of BEVs (70%).

Vehicle type	Technology progress[b]	Productionn year[c]				
		2020	2025	2030	2035	2050
ICEV (L/km)	Low	7.9	7.0	6.1	5.4	4.8
	Average	7.9	6.1	5.4	4.7	4.2
	High	7.9	5.3	4.7	4.1	3.5
HEV (L/km)	Low	4.9	4.5	4.2	4.0	3.5
	Average	4.9	4.1	3.7	3.5	3.1
	High	4.9	3.7	3.3	3.1	2.6
BEV[a] (kWh/km)	Low	0.17	0.16	0.14	0.13	0.12
	Average	0.17	0.14	0.13	0.12	0.11
	High	0.17	0.13	0.12	0.11	0.10

a. Assume BEV with 400-mile range
b. " Low" is the business-as-usual scenario, " High" is the aggressive technology advancement scenario
c. These various technologies were assessed for six different timeframes: laboratory years 2015 (reference), 2020, 2025, 2030, and 2045. A delay of 5 years was assumed between laboratory year and the production year, so the starting year for the estimates was 2020.

Text S4. Energy mix and diurnal electricity supply–demand pattern in 2050

Figure S8 shows the pattern of intraday electricity supply and demand in 2012 (the base year of PM2.5EI) and 2050 assuming changes in the power supply mix in the future. The base demand (solid black line) is based on the pattern of July 27, 2012, the day with the maximum electricity demand in 2012 (data available at Japan Atomic Energy Relations Organization (JAERO)'s website :https://www.ene100.jp/zumen/1-2-10, last accessed: 22 January 2023). In 2012, 89% of the total electricity demand was met by thermal power (Figure S8a) (METI, 2019). The power supply mix in 2050 was derived assuming that the following two targets of the Japanese government's Green Growth Strategy for carbon neutrality in 2020 would be met.

- In 2050, 50%–60% of the electricity demand will be supplied by renewables, and 30%–40% by nuclear power and thermal power with CO_2 capture, utilization, and storage (CCUS) (Cabinet, 2021).
- The solar photovoltaic (PV) capacity in 2050 will be 260 GW (METI, 2021).

The hourly generation patterns (using data from Renewable Energy Institute (REI)' s website: https://www.renewable-ei.org/en/statistics/electricity/#demand, last accessed: 22 January 2023) for each power generation type in 2012 were corrected to meet the above. However, REI did not provide data for 2012, so we referred to the 2016 intraday pattern, which is the most historical data available. As a result, it was estimated that, in 2050, 50% of the total electricity generation would be provided by renewable energy, 34% by nuclear power, and 16% by thermal power (Figure S8b).

The change in air pollutant (e.g., NO_x and SO_2) emissions due to the introduction of CCUS in thermal power plants is strongly depends on the type of CO_2 capture technology employed. Basically, SO_2 emissions will be very low, NO_x emissions will strongly depend on capture and conversion technologies and additional NO_x mitigation measures to be installed, and NH_3 emissions are expected to increase significantly (EEA, 2011). However, considering uncertainties in those estimates and uncertain factors such as future progress in denitration and desulfurization technologies, it is difficult to estimate future emission factors for thermal power plants. Therefore, the emission factor for thermal power remaining in 2050 was assumed to be the same as base year in this case. In the 2050R&E&L experiment, the power plant emission factor was corrected by a

factor of 0.18 compared to the BASE experiment since thermal power generation was reduced by 82% compared to 2012.

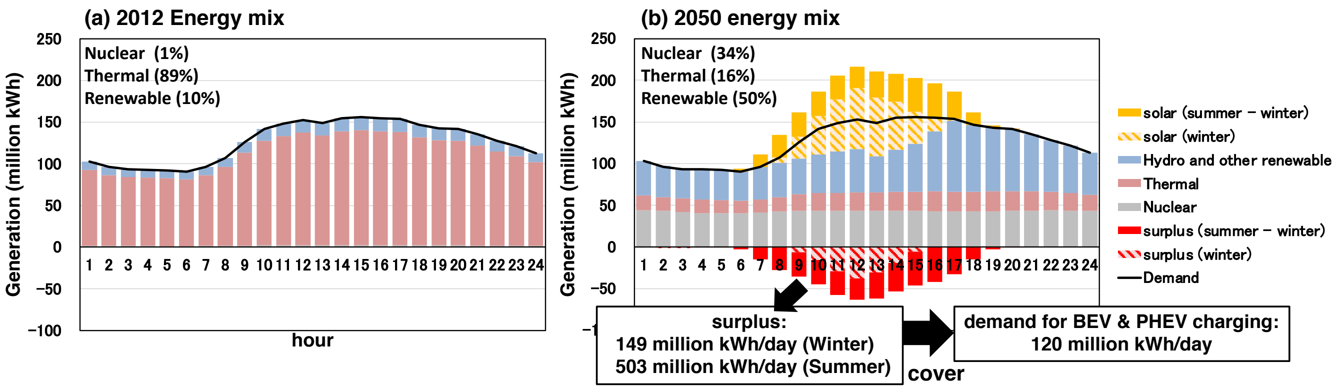

Figure S8. Diurnal variation in electricity demand and supply based on energy mix in (a) 2012 and (b) 2050. The baseline demand is the pattern of 27 July 2012, the day of the maximum electricity demand generation in 2012. The energy mix is based on the comprehensive energy statistics (METI, 2019) for 2012. For 2050, they were derived using the method described in Text S2.

Text S5. Surplus electricity of solar energy and electricity demand for BEVs and PHEVs charging in 2050

The external charging electricity for BEVs and PHEVs is mainly charged by late-night electricity at the current low rate of renewable energy adoption (MLIT urban bureau, 2012). However, with the increase in renewable energy, it is expected that surplus power will be recharged. Since the solar power generation varies by season, we evaluated whether the surplus power could cover the demand for charging power for the winter and summer cases. In 2012, the installed PV capacity was approximately 60 GW in Japan (Institute for Sustainable Energy Policies (ISEP)'s website: https://www.isep.or.jp/jsr/2017report/chapter4/4-2, last accessed: 22 January 2023). It is estimated that PV generation in 2050 will be 400 and 755 million kWh/day in winter and summer, respectively, assuming that the efficiency of solar panels remains the same and increasing the hourly PV generation increase approximately four times (PV capacity increase rate). As a result, a surplus of 149 and 503 million kWh day^{-1} is expected to be generated in winter and summer, respectively (Figure S8b). Compared to the external charging electricity demand of BEVs and PHEVs in 2050 (120 million kWh day^{-1}; text Section 2.3.4.3), this could be met by surplus solar power. It was assumed that there would be no increase in power plant emissions from BEV and PHEV charging in 2050 since even the severe assumption of winter insolation could be met. However, in the 2050R&E experiment, assuming no vehicle lightweighting by 2050 and no improvement in electricity consumption, the additional external electricity demand will be 180 million kWh day^{-1}, which may not be covered by the surplus in the winter. However, even if this could be met by thermal power generation, there would be little impact (Only 1% lower reduction rate: –81% from 2012).

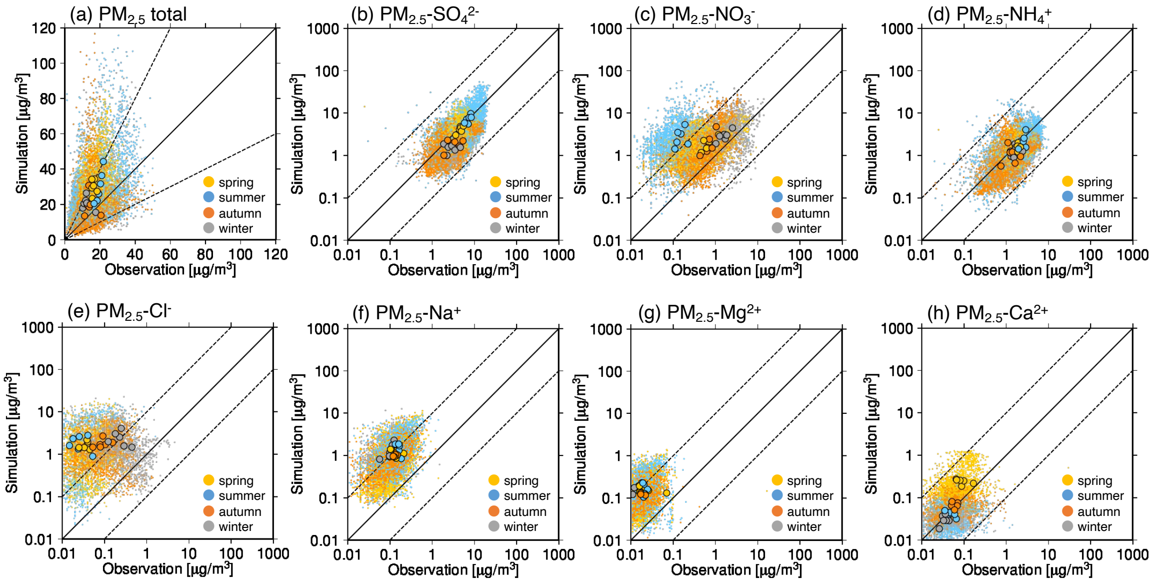

Figure S9. Scatter plots of model's BASE experiment result and observation data for (a) $PM_{2.5}$ total mass and (b–h) ion components in 2015.

The small dots are daily data (192 site nationwide × 56 days, excluding for the missing days).

The large dots are seasonal data (12 plots, 4 seasons × 3 site categories).

In (a), the solid line indicates 1:1, and the dashed lines indicate a factor of 2.

In (b–h), the solid line indicates 1:1 and the dashed lines indicate a factor of 10. For (c) NO_3^-, Simulation = a × observation + b, Observation = (Simulation – b)/a, then a and b are a = 0.84, b = 1.74 (winter), a = 1.04, b = 1.35 (spring), a = 1.98, b = 2.30 (summer), a = 2.24, b = 0.76 (autumn).

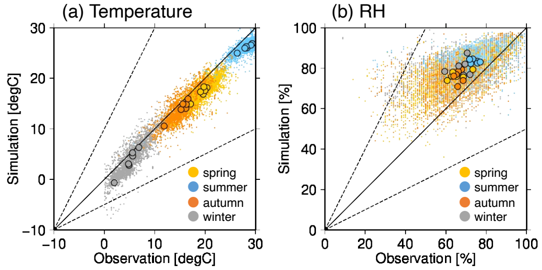

Figure S10. Same as Figure S9 but for (a) temperature and (b) RH.

For (b) RH, Simulation = a × observation + b, Observation = (Simulation – b)/a, where a and b are a = 0.38 and b = 53, respectively.

Table S7. Statistical comparison of model's BASE experiment result and observation data for the $PM_{2.5}$ total mass and that of ion components (SO_4^{2-}, NO_3^-, NH_4^+, Cl^-, Na^+, Mg^{2+}, and Ca^{2+}) in 2015.

Species	Season	N	Obs. mean (ng/m^3)	Sim. mean (ng/m^3)	R	MB (ng/m^3)	NMB (%)
$PM_{2.5}$ total mass	annual	8909	16.0	25.8	0.39	9.8	61
	winter	2118	12.5	20.8	0.39	8.3	66
	spring	2238	16.4	30.6	0.45	14.2	87
	summer	2264	20.3	31.7	0.31	11.3	56
	fall	2289	14.5	20.0	0.27	5.4	37
$PM_{2.5}$-SO_4^{2-}	annual	9129	4.7	4.1	0.60	-0.6	-13
	winter	2151	2.9	1.7	0.39	-1.2	-41
	spring	2290	4.7	4.5	0.32	-0.2	-5
	summer	2324	7.5	7.9	0.54	0.4	5
	fall	2364	3.4	2.1	0.29	-1.4	-39
$PM_{2.5}$-NO_3^-	annual	9129	0.8	2.7	0.30	1.9	247
	winter	2151	1.6	3.2	0.22	1.5	94
	spring	2290	0.7	2.1	0.21	1.4	199
	summer	2324	0.2	3.2	0.17	3.1	2008
	fall	2364	0.7	2.4	0.26	1.7	234
$PM_{2.5}$-NH_4^+	annual	9129	1.7	1.7	0.44	-0.2	-9
	winter	2151	1.4	1.4	0.49	-0.2	-14
	spring	2290	1.7	1.7	0.42	-0.2	-10
	summer	2324	2.6	2.6	0.34	-0.1	3
	fall	2364	1.2	1.2	0.31	-0.2	-14
$PM_{2.5}$-Cl^-	annual	9129	0.10	1.91	0.01	1.81	1810
	winter	2151	0.27	2.30	-0.17	2.04	756
	spring	2290	0.04	1.65	0.10	1.62	4050
	summer	2324	0.03	2.00	0.23	1.97	7880
	fall	2364	0.10	1.73	0.07	1.63	1638
$PM_{2.5}$-Na^+	annual	9129	0.13	1.23	0.44	1.10	856
	winter	2151	0.10	1.32	0.31	1.22	1281
	spring	2290	0.13	1.08	0.42	0.95	739
	summer	2324	0.15	1.47	0.50	1.32	857
	fall	2364	0.13	1.07	0.56	0.93	700
$PM_{2.5}$-Mg^{2+}	annual	9129	0.02	0.16	0.02	0.14	754
	winter	2151	0.01	0.16	0.11	0.15	1623
	spring	2290	0.03	0.15	0.01	0.13	500
	summer	2324	0.02	0.18	0.27	0.16	770
	fall	2364	0.02	0.13	0.33	0.11	667
$PM_{2.5}$-Ca^{2+}	annual	9127	0.06	0.10	0.08	0.04	64
	winter	2151	0.04	0.03	0.15	-0.004	-12
	spring	2290	0.09	0.25	0.05	0.16	178
	summer	2324	0.05	0.04	0.12	-0.01	-21
	fall	2362	0.06	0.07	0.12	0.007	12

Text S6. Seasonal variations of $PM_{2.5}$-Fe, Cu, and Zn concentrations in the Kanto urban area and the contribution of continental Asia

Figure S11 shows the seasonal variation of Fe, Cu, and Zn in $PM_{2.5}$ concentrations in area A (Figure 7 black box) in the urban area including Tokyo. The solid bars on the right axis indicate the contributions of transport from continental Northeast Asia. It was derived by source receptor (SR) analysis according to the equation (2).

$$R_{i,j} = \frac{C_{BASE,i,j} - C_{Asia-20\%,i,j}}{C_{BASE,i,j}} \times 5 \times 100 \quad (2)$$

where $R_{i,j}$ is the contribution (%) of Northeast Asian anthropogenic emissions to the concentration of metal species i $(= 3; Fe, Cu, Zn)$ at receptor grid j. $C_{BASE,i,j}$ is the concentration in the base experiment (ng m^{-3}), and $C_{Asia-20\%,i,j}$ is the concentration in the case of a 20% reduction in emissions in Northeast Asia. The emissions of domain 1 were reduced by 20%, and the results were nested, while the emissions of domain 2 were calculated as in the base experiment. The contribution from the Asian continent was high for Zn, Fe and Cu in that order, and the seasonal variations associated with continental advection (high concentrations in winter and spring, low in summer) were distinct in that order. The effect of reducing domestic emissions was higher in the summer months when the continental contribution was lower (Figure S11a–S11c). There is almost no seasonal variation in emissions (Figure S12), suggesting that the seasonal variation reflects the effects of the meteorological field. Figure S13–S15 show the spatial distribution of the monthly contribution R shown with the horizontal winds. The contribution R was higher in winter (January, February, and December) due to the northwesterly monsoon and in spring (March, April, and May) due to the prevailing westerly winds. The contribution R was lower in summer (June, July, and August) due to the predominance of southerly winds under the Pacific High. The concentrations of Fe and Zn also increase in October (Figure S11a and S11c). This may be due to the westerly winds strengthening again in October, in addition that October had many anticyclonic days, precipitation was particularly low compared to previous years, and wet deposition was low (Figure S12).

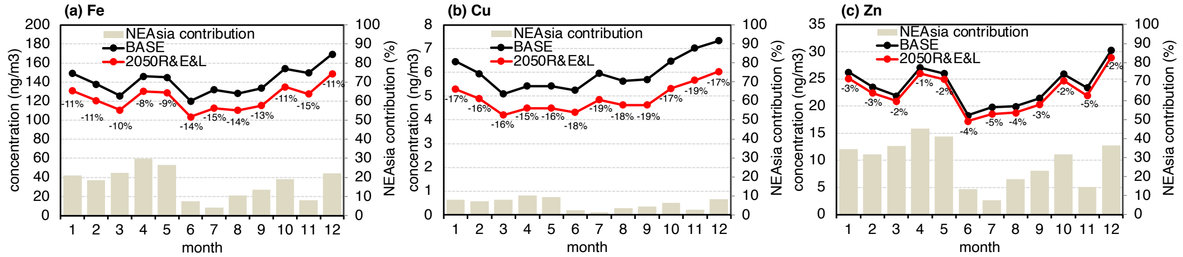

Figure S11. Seasonal variation of (a) $PM_{2.5}$-Fe, (b) $PM_{2.5}$-Cu, and (c) $PM_{2.5}$-Zn concentrations in area A (Figure 7, 139–140°E, 35–36°N). Comparison between the BASE experiment and the 2050R&E&L experiment. The numbers indicate the rate of change. The bars with the right vertical axis indicate the contribution of emissions from Northeast Asia.

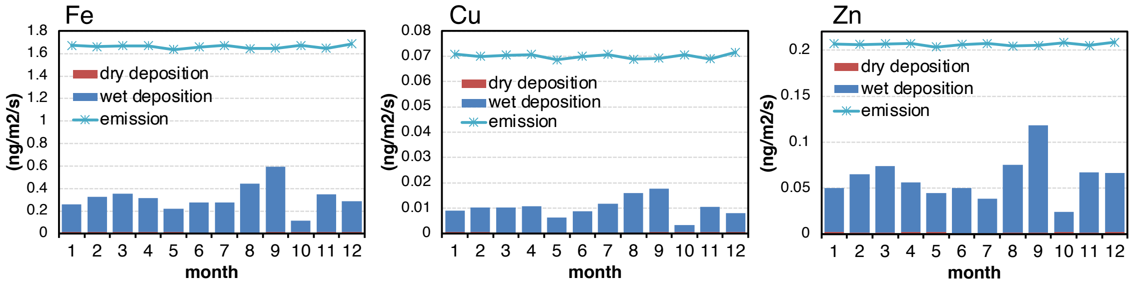

Figure S12. Seasonal variation of $PM_{2.5}$-Fe, Cu, and Zn emissions and deposition in area A (Figure 7, 139–140°E, 35–36°N).

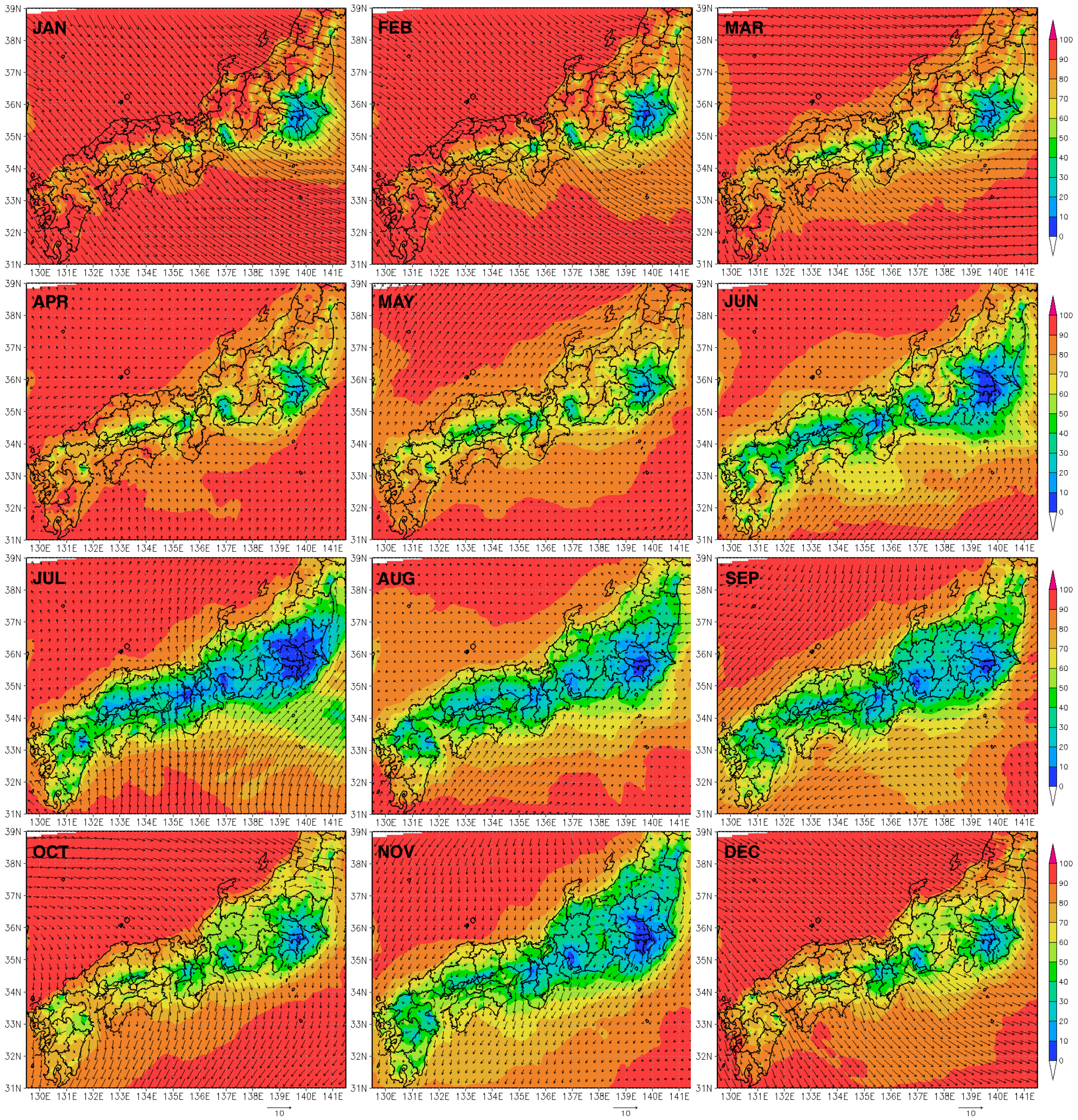

Figure S13. Contribution of continental Northeast Asian emissions to the monthly average concentration of $PM_{2.5}$ Fe derived by source–receptor analysis.
The vectors indicate the horizontal winds in the first layer of the model.

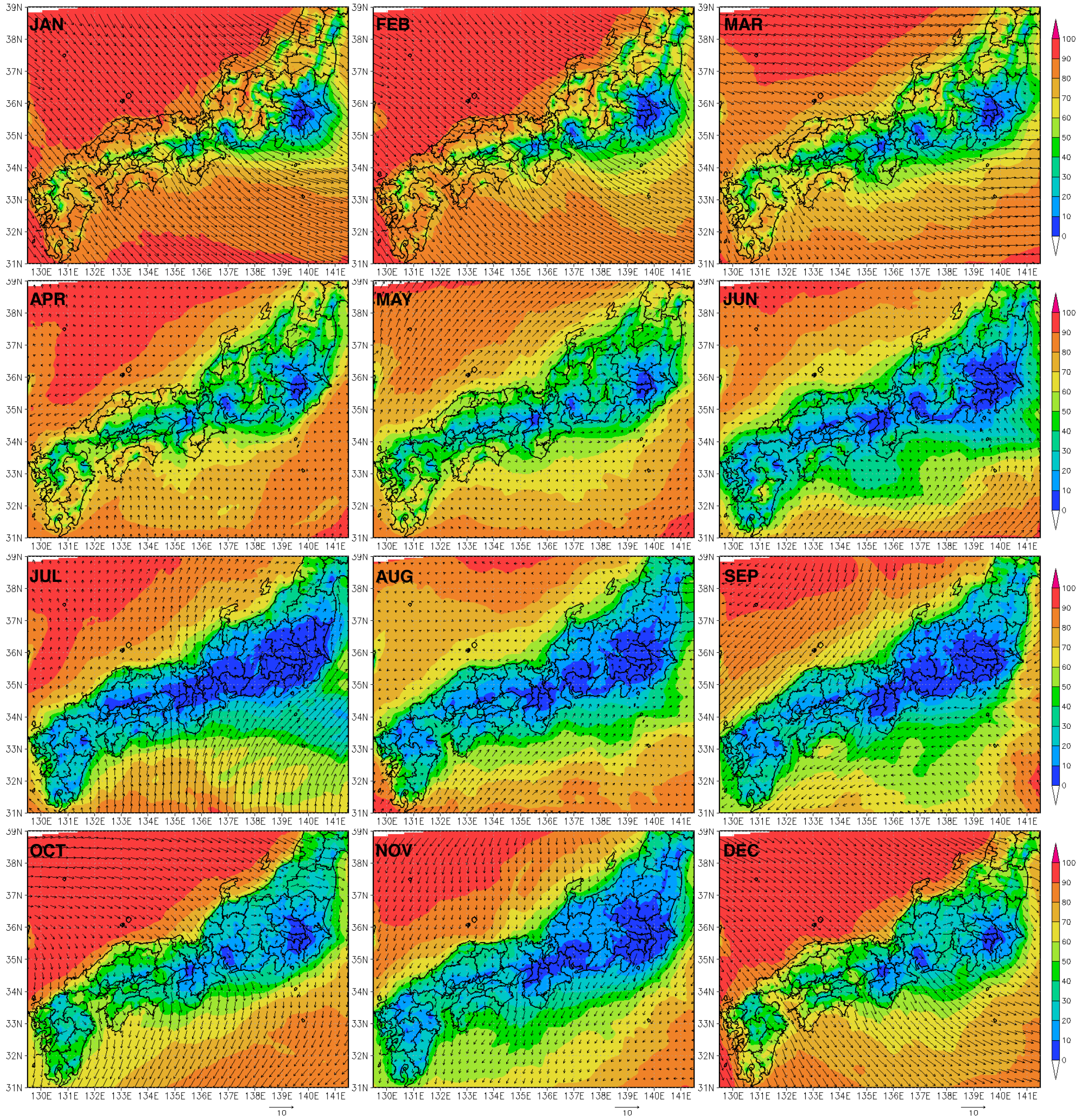

Figure S14. Same as Figure S12 but for Cu.

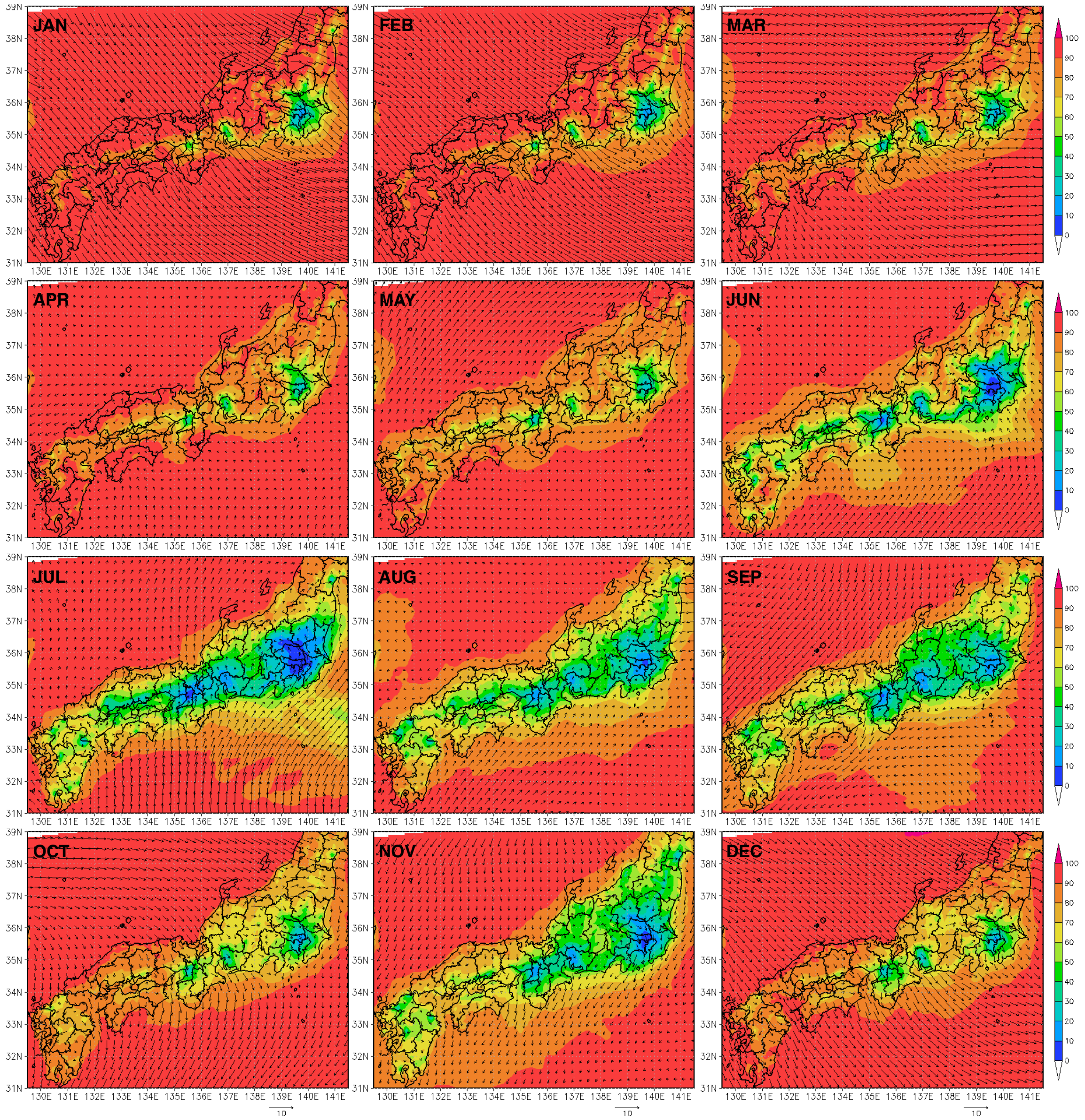

Figure S15. Same as [Figure S12](#) but for Zn.

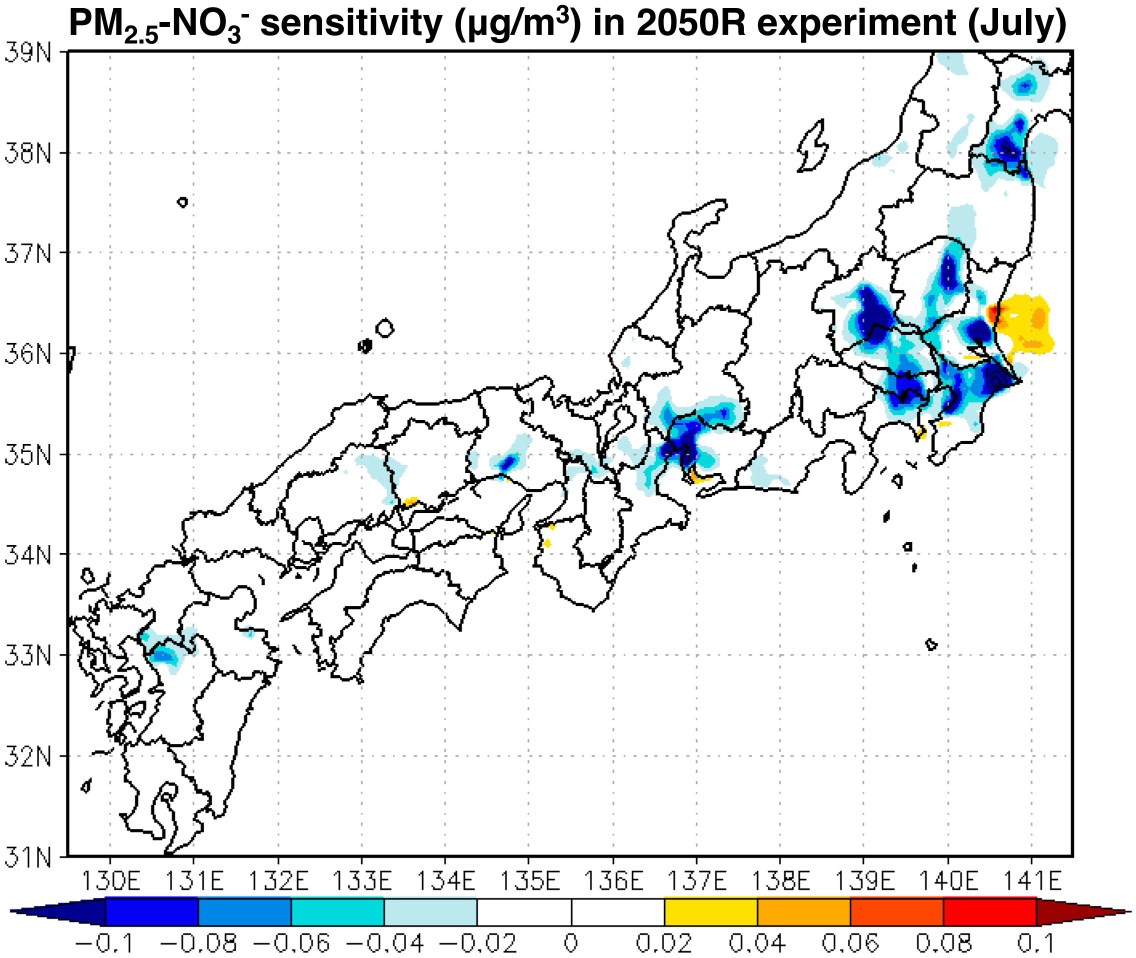

Figure S16. Sensitivity of $PM_{2.5}$-NO_3^- concentration in July by renewable energy shifting (2050R–BASE). $PM_{2.5}$-NO_3^- increased in areas near coastal thermal power plants due to the release of cations (NH_4^+, Na^+) from SO_x reduction from thermal power plants to form nitrate.

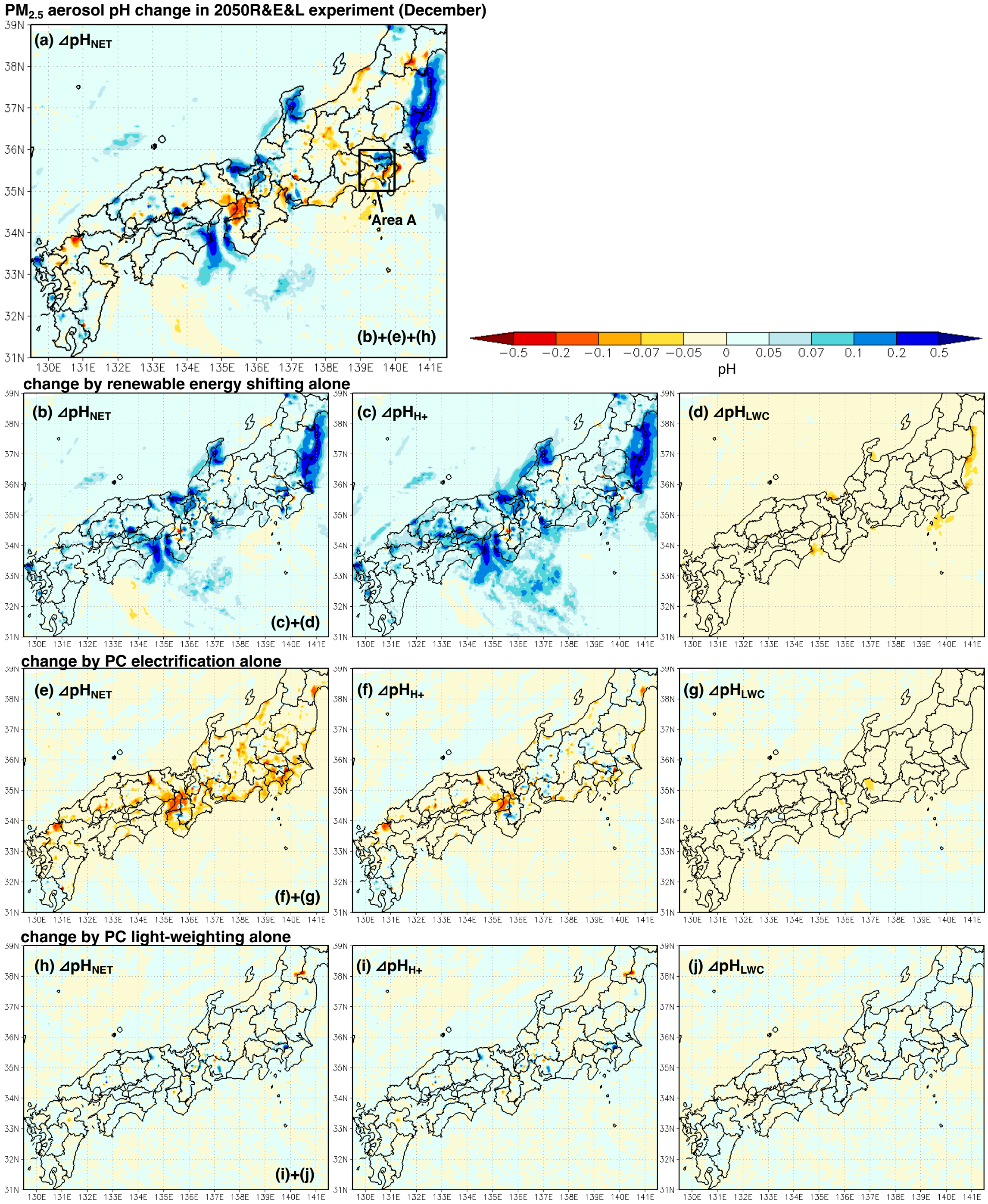

Figure S17. Same as Figure 9 but for December.

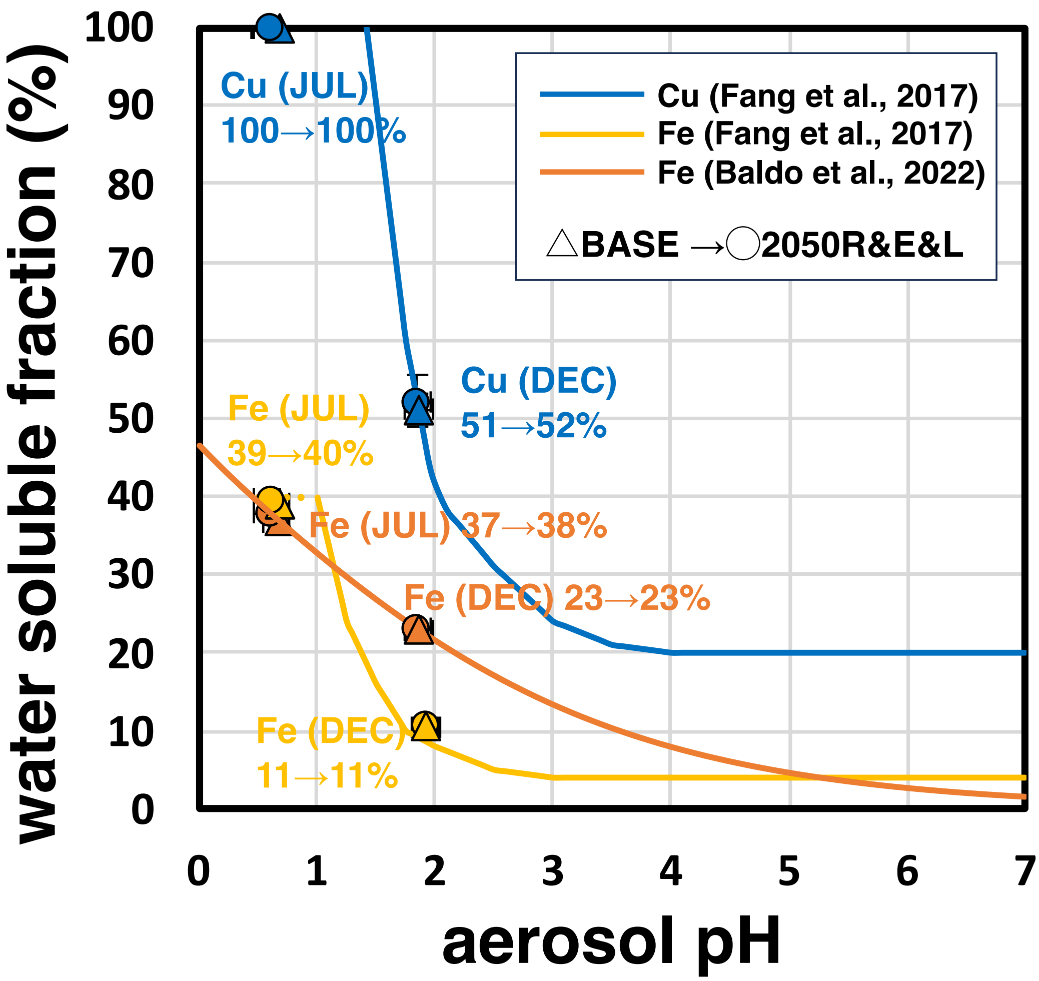

Figure S18. Relationship between aerosol pH and the water-soluble fractions of metals. The solid graph approximates the analytical results reported by Fang et al. (2017) and Baldo et al. (2022). The plots show the change in the metal solubility in the urban area (area A; Figure 7, 139–140°E, 35–36°N) due to emission changes in 2050, as estimated from the graphs. The error bars indicate the width of the model NO_3^-, RH bias (Figures S9 and Figure S10) corrected for the function of the observed values.

Fang et al (2017) collected PM in urban areas and near highways in Atlanta and indicated the relationship between the aerosol pH (calculated with the thermodynamic equilibrium model ISORROPIA-II using PM ionic composition data) and the water-soluble fraction of its metal components. Figure 2 in Fang et al (2017) was used as a reference in this study.

Baldo et al (2022) developed a model for Fe dissolution scheme using samples of coal fly ash (CFA). In this study, the proton-driven dissolution rate constant for Fe (a function of temperature and pH) provided by Baldo et al (2022) was used to derive the dissolution rate after T=298K, 48 hours.

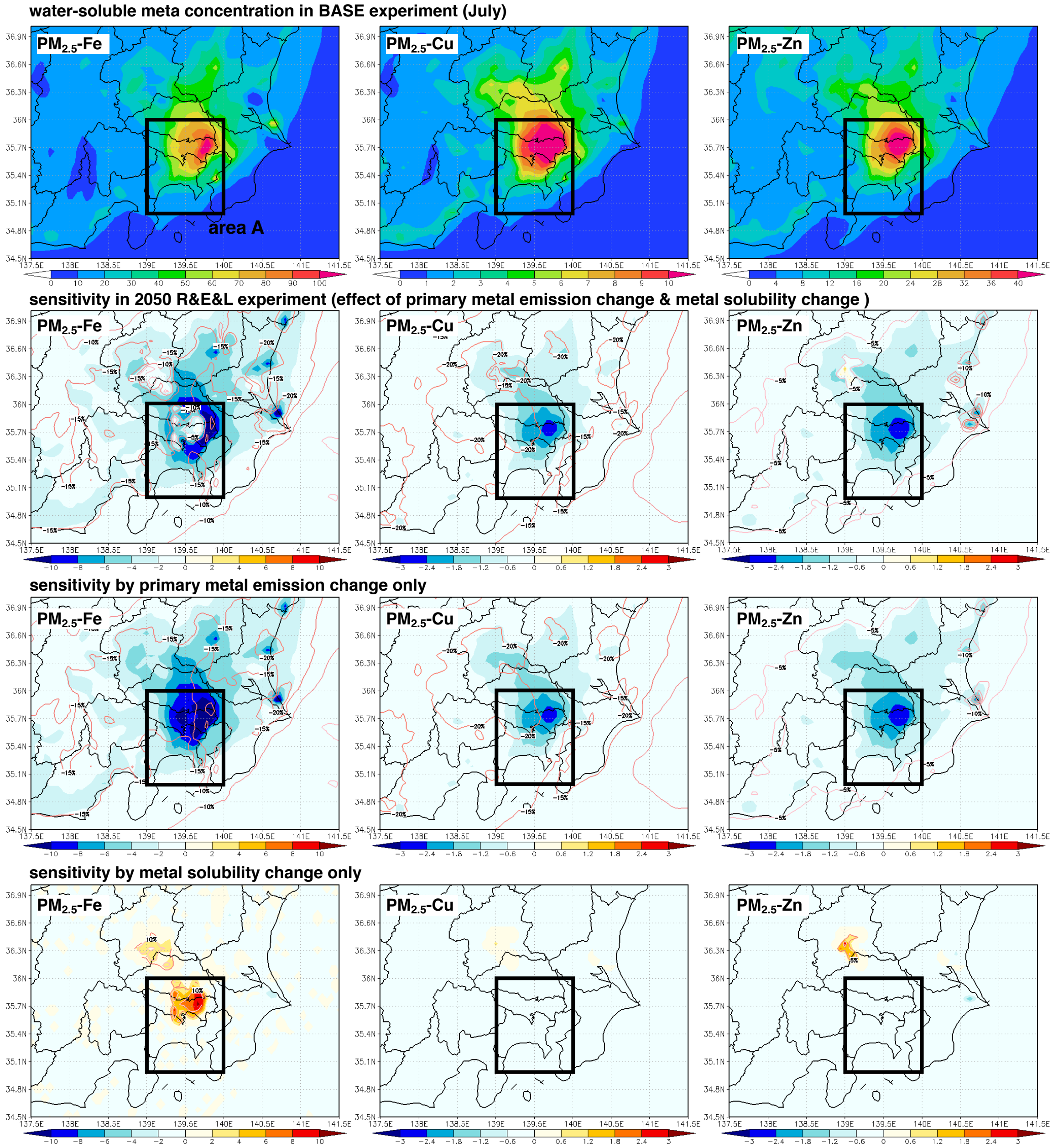

Figure S19. Concentrations of water-soluble metals in the BASE experiment in July (1st row) and sensitivity in the 2050R&E&L experiment (2nd row). The sensitivity due to changes in primary metal emissions (3rd row) and changes in aerosol acidity (4th row) alone are also indicated.
